# Histological and Somatic Mutational Profiles of Mismatch Repair Deficient Endometrial Tumours of Different Aetiologies

**DOI:** 10.3390/cancers13184538

**Published:** 2021-09-10

**Authors:** Neil A. J. Ryan, Thomas D. J. Walker, James Bolton, Natalja ter Haar, Tom Van Wezel, Mark A. Glaire, David N. Church, D. Gareth Evans, Tjalling Bosse, Emma J. Crosbie

**Affiliations:** 1 Division of Cancer Sciences, Faculty of Biology, Medicine and Health, University of Manchester, Manchester M13 9PL, UK; neilryan@nhs.net (N.A.J.R.); thomas.walker@manchester.ac.uk (T.D.J.W.); 2 Division of Evolution and Genomic Medicine, Faculty of Biology, Medicine and Health, University of Manchester, Manchester M13 9WL, UK; Gareth.Evans@mft.nhs.uk; 3 Manchester Academic Health Science Centre, Department of Pathology, Manchester University NHS Foundation Trust, Manchester M13 9WL, UK; James.Bolton@mft.nhs.uk; 4 Department of Pathology, Leiden University Medical Center, Albinusdreef 2, 2333 ZA Leiden, The Netherlands; N.T.ter_Haar@lumc.nl (N.t.H.); t.van_Wezel@lumc.nl (T.V.W.); T.Bosse@lumc.nl (T.B.); 5 Tumour Genomics and Immunology Group, Wellcome Centre for Human Genetics, University of Oxford, Roosevelt Drive, Oxford OX3 7DQ, UK; mark.glaire@gtc.ox.ac.uk (M.A.G.); david.church@well.ox.ac.uk (D.N.C.); 6 NIHR Oxford Biomedical Research Centre, Oxford University Hospitals NHS Foundation Trust, Oxford OX3 7DQ, UK; 7 Manchester Centre for Genomic Medicine, Manchester Academic Health Science Centre, Manchester University NHS Foundation Trust, Manchester M13 9WL, UK; 8 Manchester Academic Health Science Centre, Department of Obstetrics and Gynaecology, Manchester University NHS Foundation Trust, Manchester M13 9WL, UK

**Keywords:** mismatch repair, lynch syndrome, somatic mutation, endometrial cancer

## Abstract

**Simple Summary:**

Endometrial cancers can arise due to an error in DNA mending known as mismatch repair. This can happen because of an error in the cancer itself (somatic) or due to an inherited error (Lynch syndrome). Treatment trials have considered endometrial cancers caused by either of these errors as identical. As it is easier to recruit people with Lynch syndrome, they may be overrepresented in this group despite being less numerous in clinical practice. This would not be an issue if somatic and Lynch syndrome-related endometrial cancers were similar at a molecular level. The data presented herein, however, indicates that these two routes to mismatch repair, although sharing many similarities, lead to endometrial cancers with distinct molecular and pathological features. This may explain the range of outcomes observed in clinical trials of endometrial cancers with mismatch repair errors.

**Abstract:**

Background: Mismatch repair deficient (MMRd) tumours may arise from somatic events acquired during carcinogenesis or in the context of Lynch syndrome (LS), an inherited cancer predisposition condition caused by germline MMR pathogenic variants. Our aim was to explore whether sporadic and hereditary MMRd endometrial cancers (EC) display distinctive tumour biology. Methods: Clinically annotated LS-EC were collected. Histological slide review was performed centrally by two specialist gynaecological pathologists. Mutational analysis was by a bespoke 75- gene next-generation sequencing panel. Comparisons were made with sporadic MMRd EC. Multiple correspondence analysis was used to explore similarities and differences between the cohorts. Results: After exclusions, 135 LS-EC underwent independent histological review, and 64 underwent mutational analysis. Comparisons were made with 59 sporadic MMRd EC. Most tumours were of endometrioid histological subtype (92% LS-EC and 100% sporadic MMRd EC, respectively, *p* = NS). Sporadic MMRd tumours had significantly fewer tumour infiltrating lymphocytes (*p* ≤ 0.0001) and showed more squamous/mucinous differentiation than LS-EC (*p* = 0.04/*p* = 0.05). *PTEN* mutations were found in 88% sporadic MMRd and 61% LS-EC, respectively (*p* < 0.001). Sporadic MMRd tumours had significantly more mutations in *PDGFRA*, *ALK*, *IDH1*, *CARD11*, *CIC*, *MED12*, *CCND1*, *PTPN11*, *RB1* and *KRAS*, while LS-EC showed more mutations affecting *SMAD4* and *ARAF*. LS-EC showed a propensity for TGF-β signalling disruption. Cluster analysis found that wild type *PTEN* associates predominantly with LS-EC, whilst co-occurring mutations in *PTEN*, *PIK3CA* and *KRAS* predict sporadic MMRd EC. Conclusions: Whilst MMRd EC of hereditary and sporadic aetiology may be difficult to distinguish by histology alone, differences in infiltrating immune cell counts and mutational profile may predict heterogenous responses to novel targeted therapies and warrant further study.

## 1. Introduction

The Cancer Genome Atlas (TCGA) categorises endometrial cancers (EC) into four molecular subgroups that more accurately predict clinical outcomes than histological subtype [1]. Approximately one quarter are mismatch repair deficient (MMRd) [2], usually because of hypermethylation of the promoter region of *MLH1* [2], an almost exclusively somatic event [3,4]. Less commonly MMRd EC are because of Lynch syndrome (LS), an autosomal dominant hereditary condition affecting up to 3% of all EC patients [2]. MMRd tumours have an intermediate prognosis and gain reduced benefit from standard chemotherapeutic agents [5,6] but are sensitive to immune checkpoint inhibitors [7,8], with high rates of durable responses described [9]. The recent decision by the United States Food and Drug Administration (FDA) to licence immunotherapy treatments for MMRd tumours irrespective of their site of origin is, therefore, an exciting development [10]; however, the trials that informed this recommendation considered all MMRd tumours to be equal [8,11]. Sporadic and hereditary causes of MMRd reflect different underlying biology that may, in turn, influence treatment response and survival outcomes [12], and failure to account for their potential differences may be an important source of confounding [12]. This is particularly important because participants of drug registration clinical trials are predominantly those with LS-associated rather than sporadic MMRd tumours, whereas, in routine clinical practice, the reverse is true [2]. LS-associated carcinogenesis is a constant threat in individuals with inherited dysfunctional mismatch repair because DNA replication errors occur regularly during normal growth, repair and regeneration [13]. Resulting nonsense proteins, so-called frameshift peptides, are highly immunogenic, and their associated cancers are rapidly cleared by a functional immune system. Therefore, surviving cancers must develop in the context of a strong anti-cancer immune response which drives adaption and immune escape [14]. By contrast, sporadic MMRd EC develops in a mostly un-primed immune microenvironment [12]. It is, therefore, likely that sporadic and hereditary MMRd EC exploit different routes to carcinogenesis and may comprise a heterogeneous group of tumours with different biology and clinical outcomes. An improved understanding of the similarities and differences characterising MMRd EC of sporadic and hereditary origin may therefore inform therapeutic innovations, targeted treatments and personalised care.

The aim of this study was to assess pathological features and somatic mutational profiles of a large cohort of LS-EC and compare these with sporadic MMRd EC sourced from TCGA. The lack of previous studies in this area reflects historically poor routine testing of EC for MMRd, limiting the size of LS-proven tumour cohorts available for comparison with TCGA data.

## 2. Materials and Methods

Definitions:

- Hereditary MMRd: ECs in individuals with a proven germline pathogenic variant in *MLH1*, *MSH2*, *MSH6* or *PMS2*;
- Sporadic MMRd: *MLH1* hypermethylated ECs from the Nature 2014 TCGA cBioportal.

### 2.1. Tumour Selection

The Biomarkers Of Lynch syndrome Tumours (BOLT) study was sponsored by the University of Manchester and approved by the North West Greater Manchester Research Ethics Committee (ref: 16/NW/0164). Women with a germline pathogenic variant (InSiGHT Class IV or V [15] consistent with LS and a histologically confirmed diagnosis of EC were identified from two large gynaecological cancer centres in Manchester (UK) and Leiden (NL) and through collaboration with the patient support group Lynch Syndrome UK. Two formalin-fixed, paraffin-embedded (FFPE) blocks (one tumour, one normal tissue) were obtained from the hysterectomy specimen for next-generation sequencing alongside a representative haematoxylin and eosin (H&E) stained slide for pathology review. Where hysterectomy material was not available, tissue blocks and slides of the diagnostic biopsy were obtained. Sporadic MMRd EC that were microsatellite high [16] and *MLH1* hypermethylated formed the comparator cohort and were sourced from TCGA via the cBioportal (http://www.cbioportal.org/ accessed on 14 March 2020) using the Nature 2013 data.

### 2.2. Pathology Review

Tumour morphology was assessed independently by two specialist gynaecological pathologists (TB and JB) using World Health Organisation criteria [17]. Disagreements were resolved by collaborative review and discussion. Review was limited to one representative H&E slide per case to be comparable to the TCGA cohort. Pathological features of interest were histological subtype, grade, mucinous differentiation, squamous differentiation, lymphovascular space invasion (LVSI), myometrial invasion, tumour infiltrating lymphocytes (TILs) and fixation quality. Myometrial invasion was categorised according to Quick et al. [18] and LVSI extent according to Bosse et al. [19]. TILs were scored as a percentage of the stromal compartment as per Salgado et al. [20]. Fixation quality was good or poor, according to the reviewing pathologist’s opinion. The LS-EC underwent slide review, and digital images of the sporadic MMRd EC comparator cohort were reviewed on TCGA cBioportal. Pathologists were blinded to the original pathology report and each other’s report, as well as germline and somatic mutational data. Discordant cases were settled by consensus review. Tumour stage was taken from the original pathology report or TCGA cBioportal.

### 2.3. Immunohistochemistry

Immunohistochemistry was carried out on 4 μm tissue sections from representative LS-EC tumour blocks. For MMR protein immunohistochemistry, 0.3% H_2_O_2_/methanol was used to inactive endogenous peroxidases. This was followed by antigen retrieval in boiling 10 mml/L Tris-EDTA pH 9.0. Sections were incubated overnight with primary antibodies against MSH6 (clone EPR3945, 1:800, Genetex) and PMS2 (clone EP51, 1:25, Diamino-benzidine- tetrahydrochloride (DAKO)). Sections stained for PMS2 underwent incubation at room temperature with Envision FLEX + Linker (DAKO) for 20 min. All sections were subsequently incubated with a secondary antibody (poly-HRP-GAM/R/R; DPV0110HRP; Immunologic). DAKO was used as a chromogen. Sections were counterstained with Mayer’s haematoxylin, dehydrated and mounted. The proportion of stained tumour epithelial component and intensity of staining was scored by two expert independent observers using tumour stroma as internal control [21].

p53 immunohistochemistry was carried out in the Manchester University NHS Foundation Trust (MFT) Clinical Pathology Laboratory using the automated Ventana BenchMark ULTRA IHC/in situ hybridisation (ISH) staining module (Ventana Co., Tucson, AZ, USA) and ultraView 3,3’ diaminobenzidine version 3 detection system. 4 μm tissue sections were baked at 70 °C for 30 min, deparaffinised and incubated in EZPrep (Ventana Co., Tucson, AZ, USA) before washing with TRIS-based reaction buffer. Antigen retrieval used TRIS-ethylenediamine tetraacetic acid (EDTA)-boric acid buffer and cell conditioner 1 for 36 min. Sections were then incubated with ultraviolet inhibitor blocking solution for 4 min before applying DO-7 mono-clonal p53 antibody (DAKO) at 1:50 dilution for 36 min. Sections were incubated with horseradish peroxidase-linked secondary antibody, H_2_O_2_ and DAB chromogen and copper for 8, 8 and 4 min, respectively. Slides were washed, counterstained with Harris haematoxylin, dehydrated and coverslipped. p53 staining was scored using British Association of Gynaecological Pathologists protocols by two independent observers; discordant results were resolved by collaborative review and discussion, with a senior author (JB) having the final call [22].

### 2.4. DNA Extraction and Next Generation Sequencing

Tumour DNA was obtained by core biopsy of tumour blocks from several different tumour regions and compared with core biopsies from normal tissue blocks (4 × 0.6 mm of each). Where there was less material available, DNA was extracted from tissue micro-dissected from five 10 μm slides. DNA extraction was performed on the automated VersantTissue Preparation platform (TPS, Siemens Healthcare Diagnostics, Erlangen, Germany) as previously described [23]. DNA concentration was confirmed by fluorometer (Qubit dsDNA HS, Life Technologies, Carlsbad, CA, USA), with >50 ng needed for analysis. A custom-designed AmpliSeq next-generation sequencing panel (Cancer Hotspot Panel v4) was designed to capture the genes most frequently mutated in endometrial cancer described in COSMIC [24]. The bespoke panel comprised 75 genes, 32 exomic regions and 43 hotspots (Section S2 Supplementary Materials). Primer sequences are available on request. Sequencing libraries were prepared using AmpliSeq methodology according to the manufacturer’s recommendations (Thermo Fisher, Waltham, MA, USA) using 10 ng of DNA. Libraries were sequenced on the Ion Torrent Genestudio S5 platform and a 540 chip (Thermo Fisher, Waltham, MA, USA).

### 2.5. Data Analysis

The unaligned bam files generated by the sequencer were mapped against the human reference genome (GRCh37/hg19) using the TMAP 5.0.7 software with default parameters (https://github.com/iontorrent/TS accessed on 16 June 2020). Variant calling used the Torrent Variant Caller (TVC) 5.0.2 according to the recommended somatic variant caller parameter. Integrative Genomics Viewer (IGV) was used for visual inspection of variants [25]. Unless otherwise stated, all sequences have a depth of more than 100 reads, minimum base-pair quality of 20, and minimum number of reads and variants are reported with an allele frequency of >0.25. Variants were imported into the local in-house variant database Genetic Assistant (GA), Version: 1.4.5; SoftGenetics, which assigns variant annotations, functional prediction, conservation scores and disease-associated information to each variant. A five-class system was used to categorise mutations. These were assigned through a systematic search of the literature (PubMed), general or locus-specific databases (Mycancergenome, Alamut Visual, NCBI dbSNP, NCBI ClinVar, COSMIC, Jackson laboratory database, LOVD, MD Anderson, IARC TP53 database). Class I and II variations were considered benign. Class III were variations of unknown significance; it was not possible to define the downstream effect on protein function. Class IV and V were considered pathogenic. Only pathogenic/driver mutations were included in the analysis. Copy number variants (Gains, amplification, or deletions and LOH) were studied with an in-house developed copy number variation analysis tool, visualised in R (version 3.3.1) the NGSE shiny app (https://git.lumc.nl/druano/NGSE accessed on 16 June 2020). For the sporadic MMRd EC, somatic mutational data were taken directly from TCGA via the cBioportal. Only genes included in the bespoke 75-gene endometrial cancer panel used for the LS-EC were analysed.

### 2.6. Statistical Analysis

Data tidying and consolidation was conducted by VBA scripting and conditional formulae in Microsoft Excel 2010. Statistical analysis was performed using GraphPad v 7 (La Jolla, CA, USA) for comparative statistics and R 3.6.0 and RStudio programming environment for clustering analysis. FactomineR was used in addition to base R and the TidyVerse suite. For overall comparisons of percentages, Student’s t-test or ANOVA was used. Individual comparisons of percentages were carried out with the N-1 Chi-squared test [26]. For all descriptive analyses, the alpha was set at 0.05. Clinical features from the two cohorts were unified, and data matrices constructed for the 75-gene panel. Four matrices were populated and scored based on (1) a binary “presence/absence” of any mutation; (2) an ordinal “Passenger/Driver” mutation type; (3) an ordinal “Missense/In-frame/Truncation” mutation type; and (4) an ordinal “Ranked Severity” scoring system that integrated Passenger/Driver status with Mutation Type. Multiple Correspondence Analysis (MCA) [27] was employed to project the binary or ordinal mutation scorings into low-dimension Euclidean space to determine whether mutation signatures predict disease grade, disease stage, histological subtype, squamous differentiation, or mucinous differentiation. The four matrices were subsetted by LS status to provide additional four matrices for LS only analyses. These eight datasets formed the basis of bioinformatics analysis. No more than five patient outliers were removed for any MCA analysis (and no more than three during a single iteration). If necessary, the four lowest informative genes were removed prior to final MCA output. Dendrogrammatic clustering was used to assess for mutational substructure in LS and spontaneous MMRd cohorts by standardising and scaling mutation scorings.

## 3. Results

### 3.1. Pathological Features

**Figure 1:**
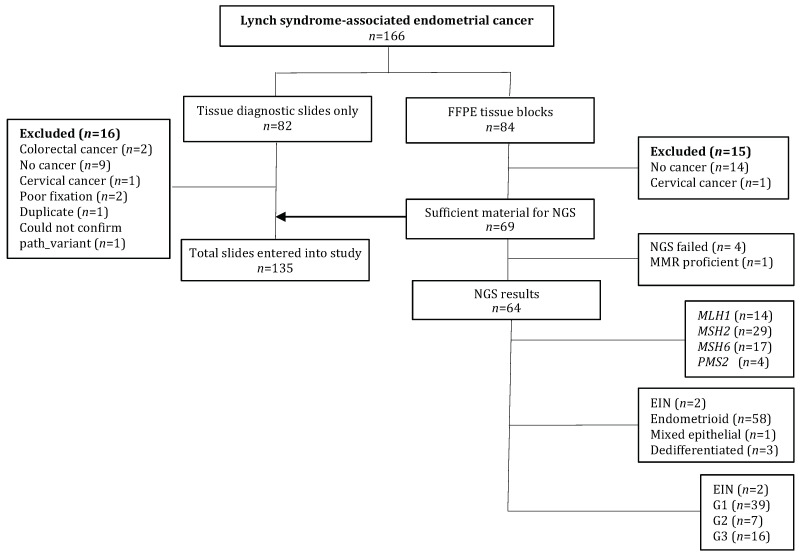
Study flow diagram Abbreviations: EIN: endometrial intraepithelial neoplasia; FFPE: formalin-fixed, paraffin-embedded; G: grade; NGS: next-generation sequencing; NK: not known.

In total, 166 diagnostic slides and/or surgical specimens were received from LS-EC proven cases treated between 1982 and 2016 (Figure 1). In total, 65 sporadic MMRd EC (MSI hyper-mutated) were identified in the TCGA, six were excluded due to MSI-L (*n* = 1) or normal *MLH1* methylation status (*n* = 5); thus, 59 tumours formed the comparator cohort. Concordance for histological subtype was high between the two pathologists with an overall Cohen’s kappa of 0.82. There were no serous tumours in either group. The LS-EC cases were younger than their sporadic MMRd counterparts, showed higher TIL counts and were more likely to demonstrate broad front myometrial invasion. The sporadic MMRd tumours were exclusively of endometrioid histological subtype with a tendency towards higher grade, squamous or mucinous differentiation and LVSI (Table 1).

### 3.2. Somatic Mutations

After filtering, the mean number of mutations in the 75 genes sequenced was 4 and 5 per LS-EC and sporadic MMRd tumour, respectively (Figures S1–S3). In the 64 LS-EC tumours, there were 246 variants, of which 28%, 36% and 35% were class III, IV and V, respectively. The most common mutation type was missense (76.4%), followed by truncating (11%). The most common base pair substitution was cystine to thymine (41%) (Figures S4–S7). Two LS-EC tumours did not have a mutation detected. For the 59 sporadic MMRd tumours, 289 variants were detected. Direct mutation class comparison was not possible due to different classification methodologies; however, 54% were considered driver variants. The most common type of variant was missense (79.2%), followed by frameshift (17.7%).

**Figure 2:**
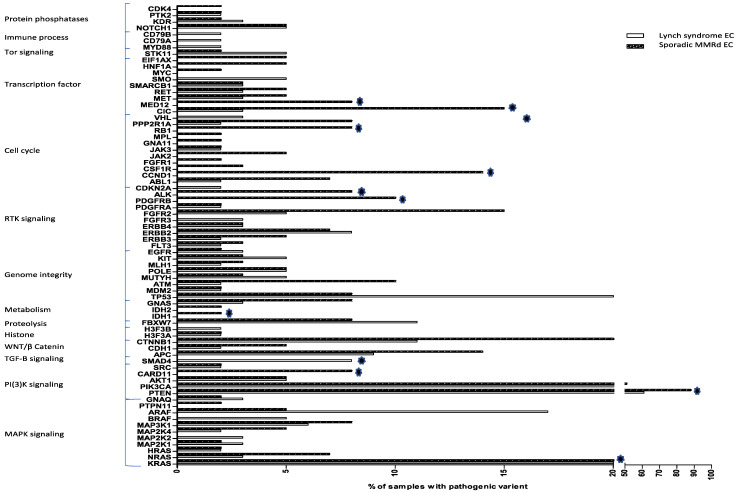
Mutational profiles of LS-EC and sporadic MMRd EC. The 75 genes included in the analysis are listed on the Y-axis grouped by associated cellular process. On the X-axis, the % of tumours with a pathogenic variant in analysed genes is demonstrated. Stars indicate genes in which the proportion of pathogenic variants between sporadic MMRd and LS-EC was significantly different.

Both LS-EC and sporadic MMRd cohorts showed a propensity for mutations in the PI(3)K and MAPK signalling pathways (Table S1). The most mutated gene was *PTEN*, seen in 61% LS-EC and 88% sporadic MMRd tumours, respectively (*p* < 0.001). *TP53* variants were seen in 20% of the LS-EC tumours but only 8% of the sporadic MMRd tumours (*p* = 0.06, NS). Mutations in *SMAD4* were significantly more common in LS-EC (8% vs. 0%, *p* = 0.04), whereas mutations in *PDGFRB* (0% vs. 10%, *p* = 0.01), *ALK* (0% vs. 8%, *p* = 0.02), *IDH1* (0% vs. 2%, *p* = 0.03), *CARD11* (0% vs. 8%, *p* = 0.02) and *KRAS* (20% vs. 39%, *p* = 0.02) were significantly more common in sporadic MMRd tumours (Figure 2). There were no mutations in genes associated with immune evasion in sporadic MMRd tumours, although our panel had limited coverage of such genes. At the oncogenic signalling pathway level, only TGF-β signalling was significantly different between the cohorts, disrupted in LS-EC tumours only (Figure 2 and Table S1).

Within the LS-EC cohort, path_*MSH6* carriers showed more mutations affecting *ARAF* and fewer mutations affecting *KRAS* than carriers of other MMR pathogenic variants (Tables S2 and S3). Only carriers of path_*MLH1* variants showed somatic mutations associated with immunological processes. Path_*MLH1* variant carriers (*n* = 14) had significantly fewer somatic mutations in *PTEN* than sporadic MMRd tumours (57% vs. 88%, *p* = 0.007). Conversely, *BRAF* (14% vs. 0%, *p* = 0.005) and *SMO* (14% vs. 0%, *p* = 0.005) were more commonly mutated in path_*MLH1* variant carriers. These data should be interpreted with caution due to low numbers of path_*MLH1* carriers.

In the LS-EC cohort, 6/11 (55%) tumours with *TP53* mutations had grade 1 disease, and a further case had endometrial intraepithelial neoplasia (EIN). By contrast, aberrant p53 expression was observed in only 5 LS-EC tumours, all with grade 2 or 3 disease. All *TP53* mutations in the sporadic MMRd cohort had grade 2 or 3 tumours. *TP53* mutations were found in 7/17 (41%) LS-EC tumours with high TIL counts. Mucinous differentiation was associated with altered PI(3)K signalling, with 13 (100%) harbouring a mutation in either *PTEN* (*n* = 6) or *PIK(3)CA* (*n* = 7). The genes not mutated in LS are shown in Tables S6 and S7.

### 3.3. Clustering Analysis

**Figure 3:**
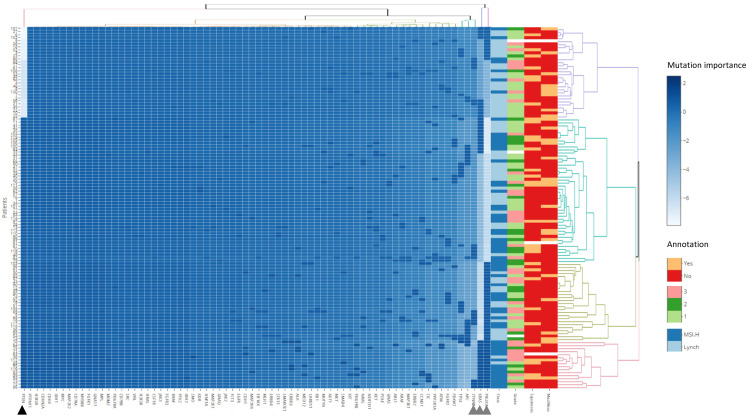
Mutational profiles. A panel of 75 genes classified by “ever mutation” for 61 LS-EC and 59 sporadic MMRd patients. Clinical annotations for class, grade, squamous and mucinous differentiation are provided as the rightmost columns. Intensities represent standardised and scaled signatures per patient observations (rows). Four principal dendrogrammatic clusters of genetic mutational signatures were observed indicative of genomic assault substructure within the pathologies. Gene clustering present mutations in *PTEN* (black triangle); *PIK3CA*, *KRAS* and *CTNNB1* (grey triangle) as the most important events in the two pathologies. Of interest, the purple dendrogram cluster shows wild type *PTEN* associates predominantly with Lynch syndrome (22 out of 30 patients; light blue “class” annotation). Co-occurring mutations in *PTEN*; *PIK3CA*, and *KRAS* predict predominantly a sporadic MMRd phenotype (15 out of 17 patients; dark blue “Class” annotation; peach dendrogram). No associations were observed between the mutational signatures and disease grade, squamous, or mucinous differentiation status.

**Figure 4:**
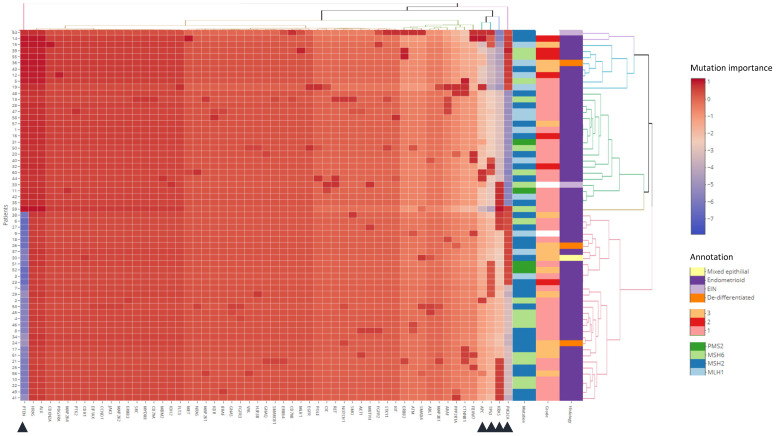
Mutation profiles within LS-EC. A panel of 56 genes classified by ‘ranked severity mutation’ for 61 LS-EC patients. Clinical annotations for Lynch pathogenic variant (*PMS2*; *MSH6*; *MSH2*; *MLH1*), grade, histological subtype are provided as the rightmost columns. Intensities represent standardised and scaled signatures per patient observations (rows). Six principal dendrogrammatic clusters of genetic mutational signatures were observed indicative of genomic assault substructure within this LS-EC cohort. Gene clustering present mutations in *PTEN*, *PIK3CA*, *KRAS*, *TP53*, and *APC* (triangles) as the most important events in the gene target panel for patients with Lynch Syndrome. No subclusters were found to associate with Lynch genotype, disease grade, or histology.

Dendrogrammatic clustering revealed mutations in *PTEN*, *PIK3CA*, *KRAS* and *CTNNB1* as the most important mutational events. Wild type *PTEN* associates predominantly with LS, whilst co-occurring mutations in *PTEN*, *PIK3CA* and *KRAS* predict sporadic MMRd (Figure 3). No associations were observed between disease grade, squamous or mucinous differentiation and mutational profile. Within the LS-EC cohort, *PTEN*, *PIK3CA*, *KRAS*, *TP53* and *APC* mutation status were the most important mutational events (Figure 4). No subclusters are associated with pathological features, possibly due to class imbalances within histology and grade. There was also no association of subclusters with LS MMR genotype, suggesting that the gene panel is positioned downstream of MMR functional ablation. Further analysis is demonstrated in Figure S8–S11.

## 4. Discussion

All MMRd cancers are considered equal in treatment trials [8,11,28] despite a lack of evidence for this assumption. We sought to explore the validity of the assumption by comparing the genotypic and phenotypic characteristics of a large cohort of proven LS-EC with sporadic MMRd endometrial tumours from TCGA. All sporadic MMRd and most LS-EC tumours were of endometrioid histological subtype. Sporadic MMRd tumours had significantly fewer tumour infiltrating lymphocytes and showed more squamous/mucinous differentiation than LS-EC. There were similar mutational landscapes in MMRd tumours regardless of aetiology, although co-occurring mutations in *PTEN*, *PIK3CA* and *KRAS* were more common in sporadic MMRd and perturbations of TGF-β signalling more common in LS-EC. Our comprehensive interrogation of the phenotype and genotype of MMRd EC of different aetiologies revealed many shared features; however, differences in immune landscapes and mutational profiles may predict heterogeneous responses to treatment and divergent clinical outcomes. Future clinical trials should consider subgroup analysis of women with MMRd tumours of hereditary and sporadic aetiology to investigate this further.

The association between endometrioid histological subtype and MMRd endometrial tumours is well established [29]; however, few previous studies have reviewed such a large bank of proven LS-EC and looked for similarities and differences with sporadic MMRd endometrial tumours. The striking difference in infiltrating immune cells between MMRd tumours of hereditary and sporadic aetiology is consistent with previous work [12] and supports the concept of tumour evolution in the context of longstanding immune pressures in LS-EC [30]. Our observation that LS-EC, but not sporadic MMRd EC, was associated with disruption of immune signalling pathways lends further support to this theory. The immunological landscape plays a crucial role in determining tumour fate, response to treatment and survival outcomes [30]. A primed immune microenvironment may explain why women with LS-EC have better recurrence-free survival than those with *MLH1* hypermethylated endometrial tumours [31].

A previous study by Libera et al. evaluated twenty LS-EC cases and five sporadic MMRd endometrial tumours using a 16-gene sequencing panel [32]. They found an association between *KRAS* mutation and sporadic MMRd tumours and noted a preponderance of *ARID1a* pathogenic variants in their cohort. Our study builds on these findings and establishes key differences between MMRd tumours of different aetiologies, with a triad of co-occurring somatic mutations in *PTEN*, *PIKCA* and *KRAS* being a common finding only in the sporadic MMRd tumours, implicating a reliance on *MAPK* and *PI(3)K* signalling. LS-EC seems to arise independently of *PTEN* mutation, which is interesting given how common such mutations are generally and in sporadic MMRd [1,33]. *TP53* mutations were prevalent in the LS-EC cohort, a consequence of defective DNA repair and widespread genomic instability [1]. It is interesting that both *MLH1* and *PTEN* are prone to epigenetic silencing through promoter methylation, and therefore, the concordance of these two mutations may indicate a shared aetiology [34]. However, our pipeline did not capture epigenetic changes, and it is most likely that MMR dysfunction and not hypermethylation was the driving mechanism [34]. *APC* mutations were detected in both cohorts despite being uncommon in endometrial cancer [1]. Further, pathogenic *APC* mutations were found in grade 3 disease suggesting that the prevailing theory that such mutations are only present in pre-cancerous or low-grade disease does not hold in MMRd EC [35].

Our study has several key strengths. First, the LS-EC cases were all from clinically confirmed pathogenic MMR variant carriers (InSiGHT Class V https://www.insight-group.org accessed on 8 January 2021) and together comprised the largest cohort of LS-EC reported in the literature. Two expert gynaecological pathologists reviewed all morphology, using slides or digital images as appropriate, with discrepancies settled by consensus. Somatic next-generation sequencing was conducted to clinical laboratory standards with a high allele frequency of >0.25 to reduce false positives. The use of TCGA data for sporadic MMRd cases ensured robust comparison with the LS-EC cases. Limitations of the study include pathology review being restricted to one representative H&E slide per case. This was to enable a fair comparison of the LS-EC and sporadic MMRd cases, for which only one digitised slide was available on the cBioportal. Stage was taken from original pathology reports due to inequitable access to the full resected specimen across LS-EC and sporadic MMRd cases. We recognise that our cohort has a limited number of non-endometrioid tumours. This limits the application of our findings to non-endometrioid MMRd EC. However, this lack of non-endometrioid ECs is also of interest as it highlights their rarity in MMRd ECs. Recruitment through Lynch Syndrome UK favoured those who survived their EC, introducing selection bias against aggressive/non-survivable disease, however of those where stage was recorded, 19% had stage III disease, comparable with the sporadic MMRd cohort (18.6%). Our LS cohort originated from two European sites, and their generalisability to non-European populations is unclear. Sequencing formalin-fixed tissue can create artefacts, particularly when using very old samples [36]. However, recent studies have endorsed their use [37]. We have not included EC with somatic path_MMR gene mutations, which account for around 3% of all EC, whereas somatic MMR silencing through hypermethylation of the promoter region of *MLH1* accounts for 16% [2]. Therefore, our cohort represents the most clinically relevant subgroup of somatic MMRd, but it does not provide complete representation [38]. Finally, panel sequencing instead of whole-genome sequencing may miss pathogenic variants [39], and restricted sampling of a single tumour block for mutational analysis may not adequately address potential tumour heterogeneity in EC [40].

## 5. Conclusions

This is the most comprehensive comparison of proven LS-EC and sporadic MMRd endometrial tumours conducted to date. We provide detailed information about the pathological features and somatic mutational profile of a large cohort of MMRd endometrial tumours of different aetiologies. There are many similarities in pathological features and mutational landscape across tumours of sporadic and hereditary origin, with key differences in *PTEN* mutations, the immune microenvironment and disrupted immunological signalling likely reflecting different routes to carcinogenesis. These differences may underlie differential treatment responses and clinical outcomes across the two groups.

## Figures and Tables

**Table 1:** Demographic and pathological features of the LS-EC and sporadic MMRd EC cohorts.

	LS-EC Total Cohort (*n* = 135)	LS-EC NGS Cohort (*n* = 64)	Sporadic MMRd EC (*n* = 59)	LS-EC (Total Cohort) vs. Sporadic MMRd EC *p*-Value
**Age at diagnosis in years (SEM)**	53 (3.1)	53 (2.1)	62 (1.2)	<0.0001
**Path_MMR variant**				
path_*MLH1*	29 (21.8%)	14 (21.9%)	NA	NA
path_*MSH2*	50 (36.1%)	29 (45.3%)	NA	NA
path_*MSH6*	43 (32.3%)	17 (26.6%)	NA	NA
path_*PMS2*	13 (9.8%)	4 (6.3%)	NA	NA
**Histology**				
Endometrioid	125 (92%)	58 (91%)	59 (100%)	<0.0001
Clear cell	1 (0.8%)	0	0	NA
Undifferentiated	4 (3%)	3 (4.7%)	0	NA
Mixed epithelial	1 (0.8%)	1 (1.5%)	0	NA
EIN	4 (3%)	2 (3%)	0	NA
**Grade**				
1	87 (64.7%)	39 (60.9%)	17 (28.8%)	<0.0001
2	19 (13.5%)	7 (10.9%)	19 (32.2%)	0.0025
3	25 (18.8%)	16 (25%)	23 (39%)	0.0029
EIN	4 (3%)	2 (3%)	0	NA
**Stage**				
I	47 (34.8%)	26 (40.6%)	41 (69.5%)	0.0006
II	3 (2.3%)	1 (1.5%)	4 (6.8%)	0.13
III	11 (8.2%)	7 (11%)	11 (18.6%)	0.19
IV	0	0	3 (5.1%)	NA
EIN	4 (3%)	2 (3%)	0	NA
Not known	70 (51.9%)	28 (43.7%)	0	NA
**Myometrial invasion**				
Broad front	66 (48.1%)	31 (48.4%)	15 (25.4%)	0.003
Infiltrating gland	19 (14.3%)	13 (20.3%)	12 (20.3%)	0.3
MELF	4 (3%)	3 (4.7%)	0	NA
Adenomyosis invasion	3 (2.3%)	2 (3%)	0	NA
Non-specific invasion	16 (12%)	4 (6%)	0	NA
No invasion/superficial	21 (15.8)	9 (14.1%)	28 (47.5%)	<0.0001
Not known/not applicable *	6 (4.5%)	2 (3%)	4 (6.8%)	0.51
**LVSI**				
Present	13 (9.8%)	4 (6%)	12 (20.3%)	0.047
Significant	8 (6%)	7 (10.9%)	3 (5.1%)	0.81
Absent	108 (79.7%)	51 (79.7%)	41 (69.5%)	0.13
Not known/not applicable *	6 (4.5%)	2 (3%)	3 (5.1%)	0.86
**TILs ^**				
>80%	33 (24.8%)	19 (29.7%)	1 (1.7%)	<0.0001
51–80%	50 (36.1%)	22 (34.4%)	13 (22%)	0.05
11–50%	33 (24.8%)	18 (28.1%)	26 (44.1%)	0.56
0–10%	12 (9%)	3 (4.5%)	18 (30.5%)	<0.0001
Not known/Not applicable *	7 (5.3%)	2 (3%)	1 (1.7%)	0.13
**Squamous differentiation**	25 (18.8%)	13 (20.3%)	19 (32.2%)	0.042
**Mucinous differentiation**	22 (16.5%)	12 (18.75%)	17 (28.8%)	0.051

Abbreviations: SEM: standard error of the mean; LS: Lynch syndrome; EC: endometrial cancer; NGS: next-generation sequencing; MMRd: mismatch repair deficiency; path: pathological variant; EIN: endometrioid intraepithelial neoplasia; MELF: microcystic elongated and fragmented; LVSI: lymphovascular space invasion; TILS: tumour infiltrating lymphocytes * Pre-surgical biopsy or EIN sample. ^ One participant had a concurrent infection and was excluded from TILs analysis. # EIN not included (*n* = 2).

## Data Availability

Mutational data is available on request.

## Supplementary Materials

The following are available online at https://www.mdpi.com/article/10.3390/cancers13184538/s1, Table S1: A percentage breakdown of onco-genic pathways affected in LS vs. MSI-H *MLH1* methylated samples; Table S2: Somatic mutations in genes covered by The Leiden Endometrial onco- panel found in LS (from de-novo NGS sequencing) and MSI-H/*MLH1* methylated (from TCGA data) ECs; Table S3: Somatic mutations in genes covered by The Leiden Endometrial onco- panel found in LS broken down by the germline pathogenic variation; Table S4: Somatic mutations in genes covered by The Leiden Endometrial onco- panel found in path_*MLH1*; Table S5: A comparison of somatic mutations in genes covered by The Leiden Endometrial onco-panel found in our LS cohort vs. the molecular cohorts as taken from the Cancer Genome Atlas; Table S6: Genes included in the analysis; Table S7: Genes with no mutations in the Lynch cohort; Figure S1: The percentage of mutations by type in the unfiltered sequencing output; Figure S2: The percentage of mutations by class in the unfiltered sequencing output; Figure S3: The percentage of mutations by class in the unfiltered sequencing output; Figure S4: The percentage base changes in the unfiltered sequencing output; Figure S5: The percentage of mutations by type in the filtered sequencing output.; Figure S6: The percentage of mutations by class in the filtered sequencing output; Figure S7: The percentage base changes in the filtered sequencing output; Figure S8: Mutation signatures within Lynch & MSI-H patients; Figure S9: Correspondence Analysis (MCA) of Lynch Syndrome patients: Genomic Profile & Grade; Figure S10: MCA of Lynch syndrome patients: Mucinous and Squamous status; Figure S11: Contribution of gene mutations to MCA dimensions 1&2 for Lynch syndrome; Figure S12: Biplot variable contribution of gene mutations to MCA dimensions 1 & 2 for Lynch syndrome; Figure S13: Representative comparison for four MCA 3D plots for Lynch syndrome “Grade” annotation for each of the four scoring matrices.
