# Supplementary material for: Histological and Somatic Mutational Profiles of Mismatch Repair Deficient Endometrial Tumours of Different Aetiologies
Source: Cancers (Basel). 2021 Sep 10;13(18):4538. doi: 10.3390/cancers13184538 (PMC8469577; doi:10.3390/cancers13184538)
Supplement: Supplementary file 1 [file cancers-13-04538-s001.zip › cancers-1362546-supplementary.pdf]

The characterization of driver somatic mutations in Lynch syndrome associated endometrial cancer, with the use of a novel cancer hotspot next-generation sequencing panel in multiple histotypes.

Supplementary materials

Table of Contents

1 Immunohistochemistry Protocols 3

2 NGS Assay characteristics 4

3 Characteristics of mutations for the LS cohort 7

4 Somatic Landscape by path_MMR gene 14

5 A comparison of somatic landscape between path_*MLH1* vs somatic MSI-H *MLH1* promoter hyper-methylation 15

6 Somatic landscape of Lynch cohort vs TCGA derived molecular cohorts 16

7 Effect of type of mutation: driver vs passenger 17

List of Figures

Figure S1 The percentage of mutations by type in the unfiltered sequencing output....7
Figure S2 The percentage of mutations by class in the unfiltered sequencing output....8
Figure S3 The percentage of mutations by class in the unfiltered sequencing output....9
Figure S4 The percentage base changes in the unfiltered sequencing output....10
Figure S5 The percentage of mutations by type in the filtered sequencing output. 11
Figure S6 The percentage of mutations by class in the filtered sequencing output. 12
Figure S7 The percentage base changes in the filtered sequencing output....13
Figure S8 Mutation signatures within Lynch & MSI-H patients....20
Figure S9 Correspondence Analysis (MCA) of Lynch Syndrome patients: Genomic Profile & Grade....22
Figure S10 MCA of Lynch syndrome patients: Mucinous and Squamous status 24
Figure S11 Figure S11 Contribution of gene mutations to MCA dimensions 1&2 for Lynch syndrome....25
Figure S12 Biplot variable contribution of gene mutations to MCA dimensions 1 & 2 for Lynch syndrome....27
Figure S13 Representative comparison for four MCA 3D plots for Lynch syndrome "Grade" annotation for each of the four scoring matrices....29

List of Tables:

Table S1 A percentage breakdown of onco-genic pathways affected in LS vs. MSI-H MLH1 methylated samples 5
Table S2 Somatic mutations in genes covered by The Leiden Endometrial onco-panel found in LS (from de-novo NGS sequencing) and MSI-H/MLH1 methylated (from TCGA data) ECs....6
Table S3 Somatic mutations in genes covered by The Leiden Endometrial onco-panel found in LS broken down by the germline pathogenic variation....14
Table S4 Somatic mutations in genes covered by The Leiden Endometrial onco-panel found in path_MLH1....15
Table S5 A comparison of somatic mutations in genes covered by The Leiden Endometrial onco-panel found in our LS cohort vs. the molecular cohorts as taken from the Cancer Genome Atlas....16
Table S6 Genes included in the analysis....17
Table S7 Genes with no mutations in the Lynch cohort....18

1 Immunohistochemistry Protocols

Immunohistochemistry was carried out on 4µm tissue sections from representative LS-EC tumour blocks.

For MMR protein immunohistochemistry, 0.3% H_20_2/methanol was used to inactive endogenous peroxidases. This was followed by antigen retrieval in boiling 10mml/l Tris-EDTA pH 9.0. Sections were incubated overnight with primary antibodies against MSH6 (clone EPR3945, 1:800, Genetex) and PMS2 (clone EP51, 1:25, DAKO). Sections stained for PMS2 underwent incubation at room temperature with Envision FLEX+ Linker (Dako) for 20 minutes. All sections were subsequently incubated with a secondary antibody (poly-HRP-GAM/R/R; DPV0110HRP; Immunologic). Diamino-benzidine- tetrahydrochloride (DAKO) was used as a chromogen. Sections were counterstained with Mayer's haematoxylin, dehydrated and mounted.

p53 immunohistochemistry was carried out in the Manchester University NHS Foundation Trust (MFT) Clinical Pathology Laboratory using the automated Ventana BenchMark ULTRA IHC/in situ hybridisation (ISH) staining module (Ventana Co., Tucson, AZ, USA) and ultraview 3,3' diaminobenzidine version 3 detection system. 4µm tissue sections were baked at 70°C for 30 minutes, deparaffinised and incubated in EZPrep (Ventana Co.) before washing with TRIS-based reaction buffer. Antigen retrieval used TRIS-ethylenediamine tetracetic acid (EDTA)-boric acid buffer and cell conditioner 1 for 36 minutes. Sections were then incubated with ultraviolet inhibitor blocking solution for 4 minutes before applying DO-7 mono-clonal p53 antibody (DAKO) at 1:50 dilution for 36 minutes. Sections were incubated with horseradish peroxidase-linked secondary antibody, H_2O_2 and DAB chromogen and copper for 8, 8 and 4 minutes respectively. Slides were washed, counterstained with Harris haematoxylin, dehydrated and cover slipped.

2 NGS Assay characteristics

With the Ampliseq Cancer Hotspot Panel v4 NGS panel, on an Ion S5, the following genes (exons in brackets) are analyzed; (n=32)

ARAF (2,4-6,7,9,10,11,15,16); *CD79B* (5.6); *CIC* (5); *CTNNB1* (1,2,4,7,8,12,15); *EIF1AX* (1.3-6); *ERBB3* (23); *KRAS* (2-4); *NRAS* (2-4); *HRAS* (2-3); *BRAF* (6.11.15); *EGFR* (3, 7, 15, 18, 21); *GNAQ* (4.5); *GNAS* (8-9); *H3F3A* (2); *H3F3B* (2); *IDH1* (4); *IDH2* (4); *KIT* (2.9-18); *MAP2K1* (2-4,6,7,11); *MAP2K2* (2.3); *MAP2K4* (2.4.5.9); *MAP3K1* (5.8,13,14,16,17,20); *MDM2* (3,4,6,7,8); *MED12* (2); *MYD88* (3b, 5), *MUTYH* (7.13); *PDGFRA* (12, 14, 15, 18, 23); *PDGFRB* (12.14); *PIK3CA* (2.5, 6-10, 14, 18, 21); *POLE* (9-14); *RET* (10-12, 15, 16); *TP53* (1-11).

Hotspots are also analyzed in the following genes (n=43):
ABL1; AKT1; ALK; APC; ATM; CARD11; CD79A; CDK4; CDH1; CDKN2A; CSF1R; CTNNB1; ERBB2; ERBB4; EZH2; FBXW7; FGFR1; FGFR2; FGFR3; FLT3; FOXL2; GNA11; HNF1A; JAK2; JAK3; KDR; PP2R1A; MLH1; MET, MPL; MYC; NOTCH1; NPM1; PTEN; PTK2; PTPN11; RB1; SMAD4; SMARCB1; SMO; SRC; STK11; VHL.

Amplification / gain and deletions or LOH are studied with a CNV analysis tool. If the tumor cell percentage is lower than 40%, the analysis is less reliable. Unless otherwise stated, all sequences have a depth of more than 100 reads and variants are reported with an allele frequency of 0.05 or more. Class 1 and 2 variants are not reported. Class 3 are variants whose effect is unknown. Class 4 is a possible pathogenic variation and class 5 is a pathogenic variation. *: stop codon.

A

Pathway	Lynch (%)	Sporadic MSI-H (%)	P-Value
MAPK signalling	6	6	1
PI(3)K Signalling	20	31	0.16
TGF-B Signalling	8	0	0.03*
WNT/β-catenin	7	13	0.27
Histone	1	1	1
Proteolysis	11	8	0.57
Metabolism	1	4	0.28
Genome integrity	5	5	1
RTK signalling	3	5	0.57
Cell Cycle	1	4	0.28
Transcription factor	2	5	0.36
Tor Signalling	5	2	0.37
Immune Processes	2	0	0.28
Protein phosphatase	2	3	0.7

B

Pathway	path_*MLH1* (%)	Sporadic MSI-H (%)	P-Value
MAPK signalling	8	6	0.78
PI(3)K Signalling	20	31	0.42
TGF-B Signalling	0	0	1
WNT/β-catenin	2	13	0.24
Histone	0	1	0.71
Proteolysis	0	8	0.28
Metabolism	0	4	0.45
Genome integrity	3	5	0.75
RTK signalling	2	5	0.63
Cell Cycle	0	4	0.45
Transcription factor	4	5	0.88
Tor Signalling	7	2	0.32
Immune Processes	7	0	0.04*
Protein phosphatase	9	3	0.32

Table S1 A percentage breakdown of onco-genic pathways affected in LS vs. MSI-H MLH1 methylated samples

(A) and between path_MLH1 carriers vs. MSI-H MLH1 Methylated samples (B) as determined by mutations included in the the Leiden Endometrial onco-panel.

	Gene	LS Overall (%)	*MLH1* Methylated (%)	P-value
MAPK Signaling	*KRAS*	20	39	0.02
	NRAS	3	7	0.31
	HRAS	2	2	1
	MAP2K1	3	2	0.73
	MAP2K2	3	0	0.18
	MAP2K4	2	5	0.37
	MAP3K1	6	8	0.67
	BRAF	5	0	0.2
	ARAF	17	5	0.04
	PTPN11	0	2	0.03
	GNAQ	3	2	0.73
PI(3)K Signaling	*PTEN*	61	88	0.0007
	PIK3CA	34	51	0.06
	AKT1	5	5	1
	CARD11	0	8	0.02
	SRC	2	2	1
TGF-B Signaling	*SMAD4*	8	0	0.03
WNT/β-catenin	*APC*	9	14	0.39
	CDH1	2	5	0.36
	CTNNB1	11	20	0.17
Histone	*H3F3A*	2	2	1
	H3F3B	2	0	0.2
	EZH2	0	0	1
Proteolysis	*FBXW7*	11	8	0.58
Metabolism	*IDH1*	0	2	0.03
	IDH2	0	2	0.2
	GNAS	3	8	0.23
Genome integrity	*TP53*	20	8	0.06
	MDM2	2	2	1
	ATM	2	10	0.06
	MUTYH	5	3	0.58
	POLE	5	5	1
	MLH1	2	3	0.7
	KIT	5	3	0.58
	EGFR	3	2	0.73
Protein phosphatase	*NOTCH1*	5	5	1
	KDR	3	2	0.73
	PTK2	2	2	1
	CDK4	0	2	0.31
RTK Signaling	*FLT3*	2	3	0.73
	ERBB3	2	5	0.36
	ERBB2	8	7	0.83
	ERBB4	3	3	1
	FGFR3	3	0	0.31
	FGFR2	5	15	0.067
	PDGFRA	2	2	1
	PDGFRB	0	10	0.012
	ALK	0	8	0.02
	CDKN2A	2	0	0.31
Cell Cycle	*ABL1*	2	7	0.18
	CCND1	0	14	0.002
	CSF1R	0	3	0.19
	FGFR1	0	2	0.3
	JAK2	0	5	0.085
	JAK3	2	2	1
	NPM1	0	0	1
	GNA11	0	2	0.3
	MPL	0	2	0.3
	RB1	0	8	0.02
	PPP2R1A	2	8	0.12
	VHL	3	0	0.2
Transcription factor	*CIC*	3	15	0.019
	MED12	0	8	0.02
	MET	3	5	0.58
	RET	3	5	0.57
	SMARCB1	3	3	1
	SMO	5	0	0.092
	FOXL2	0	0	1
	MYC	0	2	0.31
	HNF1A	0	5	0.085
	EIF1AX	0	5	0.07
Tor Signaling	*STK11*	5	2	0.37
Immune processes	*MYD88*	2	0	0.31
	CD79A	2	0	0.31
	CD79B	2	0	0.28

Table S2 Somatic mutations in genes covered by The Leiden Endometrial onco-panel found in LS (from de-novo NGS sequencing) and MSI-H/MLH1 methylated (from TCGA data) ECs

3 Characteristics of mutations for the LS cohort

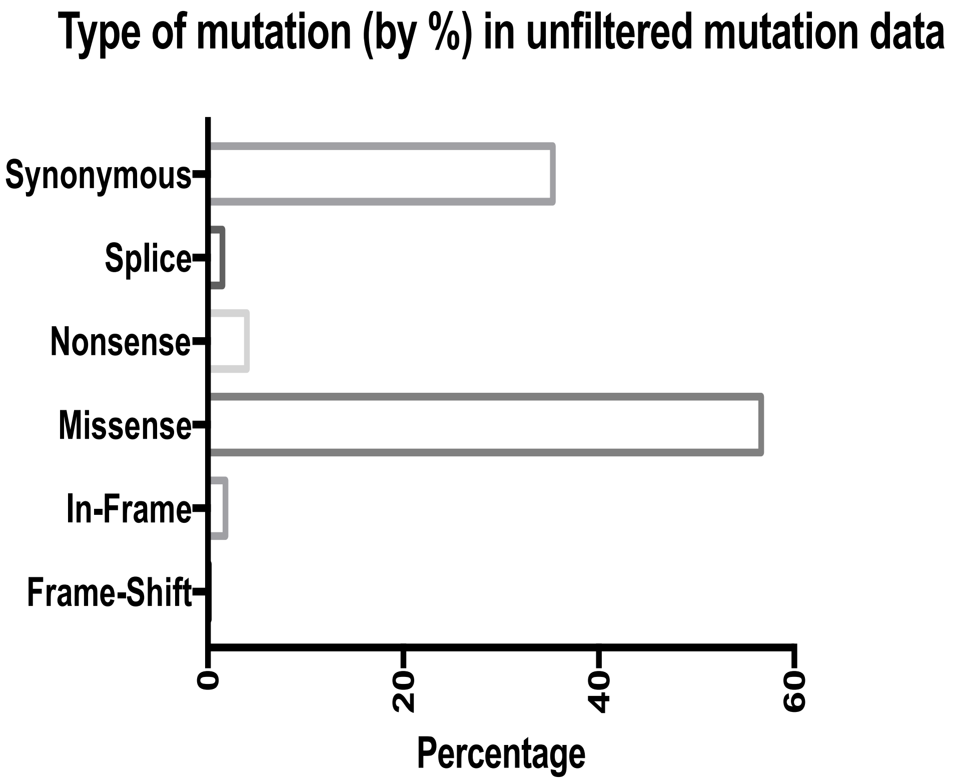

Frame-Shift	In-Frame	Missense	Nonsense	Splice	Synonymous
0.1	1.8	56.6	4	1.5	35.3

Figure S1 The percentage of mutations by type in the unfiltered sequencing output.

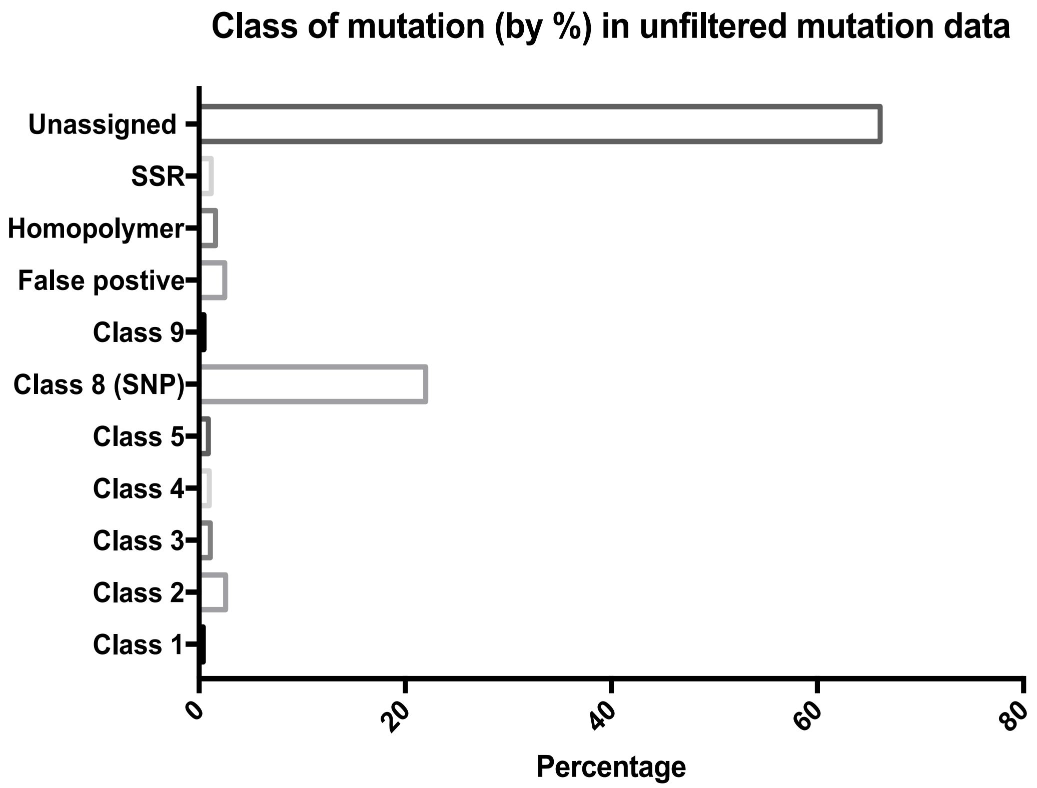

Class 1- Not Pathogenic	0.43
Class 2-Unlikely pathogenic	2.59
Class 3-Unknown pathogenicity	1.11
CLass 4-Likely pathogenic	1.01
Class 5-clinical pathogenic	0.90
Class 8-Identification SNP	22.02
Class 9-To be discussed	0.48
False Positive	2.49
Homopolymer	1.61
SSR	1.21
Unassigned	66.12

Figure S2 The percentage of mutations by class in the unfiltered sequencing output.

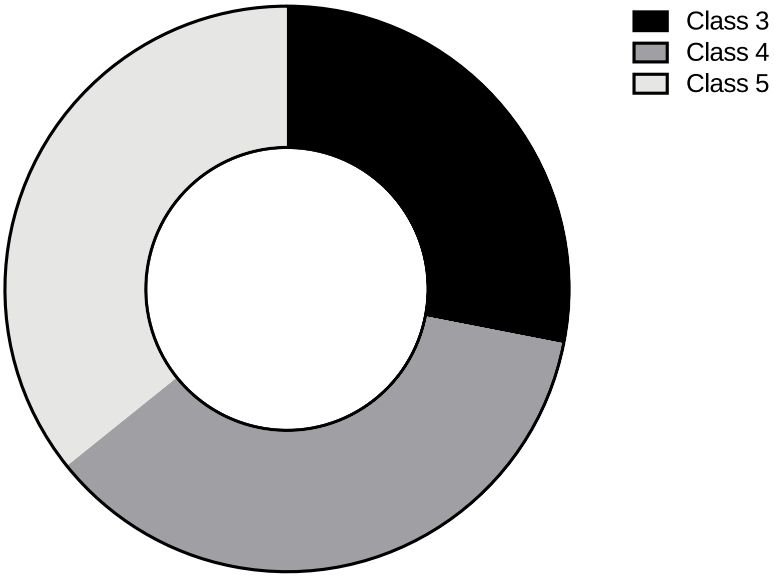

	%
Class 3	28.10
Class 4	36.20
Class 5	35.80

Figure S3 The percentage of mutations by class in the unfiltered sequencing output.

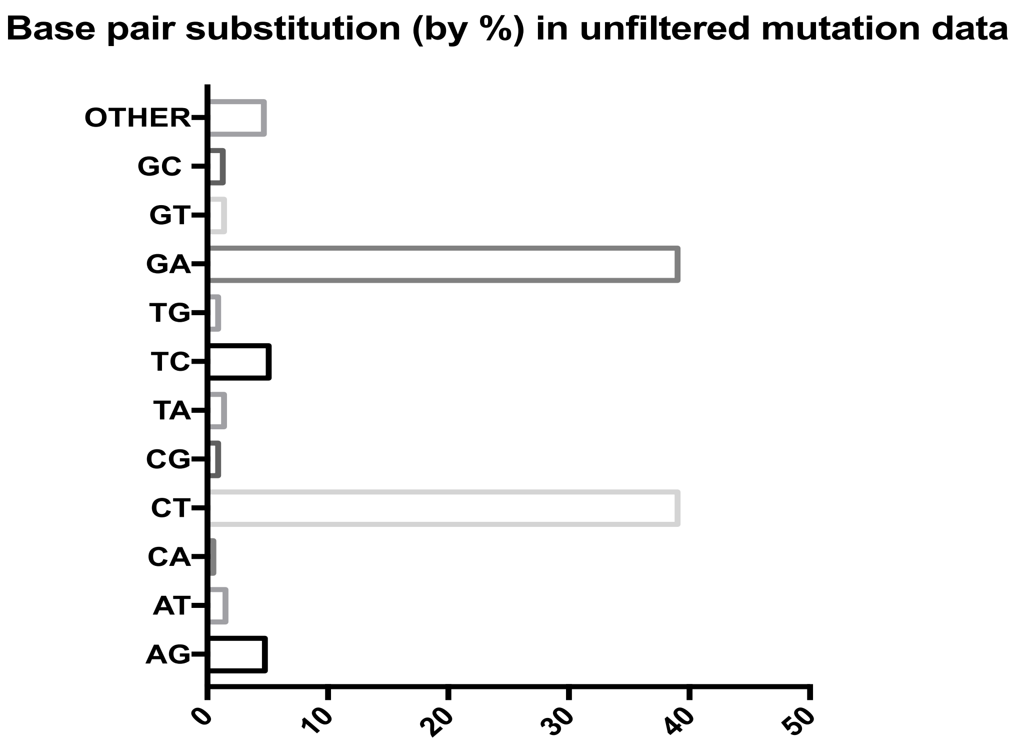

AG	4.82
AT	1.49
CA	0.45
CT	38.96
CG	0.93
TA	1.42
TC	5.14
TG	0.85
GA	38.56
GT	1.38
GC	1.30
Other	4.68

Note: Daughter strand read means this data presents includes proportionate duplicates

Figure S4 The percentage base changes in the unfiltered sequencing output.

NB: First base pair is the reference base pair and the second is the mutated base pair, therefore AG indicates adenine has been substituted with guanine.

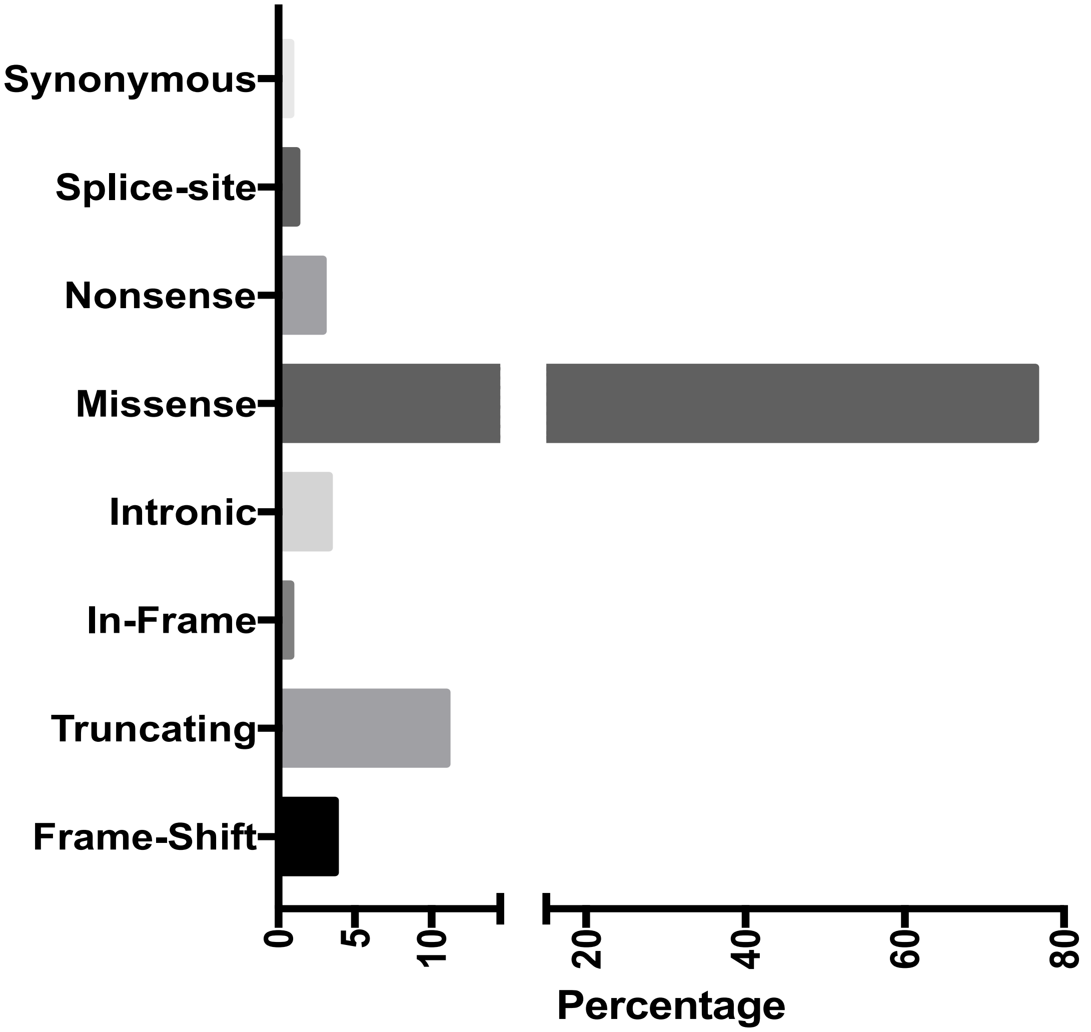

Frame-Shift	3.66
Truncating	10.97
Inframe	0.81
Intronic	3.25
Missense	76.42
Nonesense	2.85
Splice-site	1.22
Synonymous	0.81

Figure S5 The percentage of mutations by type in the filtered sequencing output.

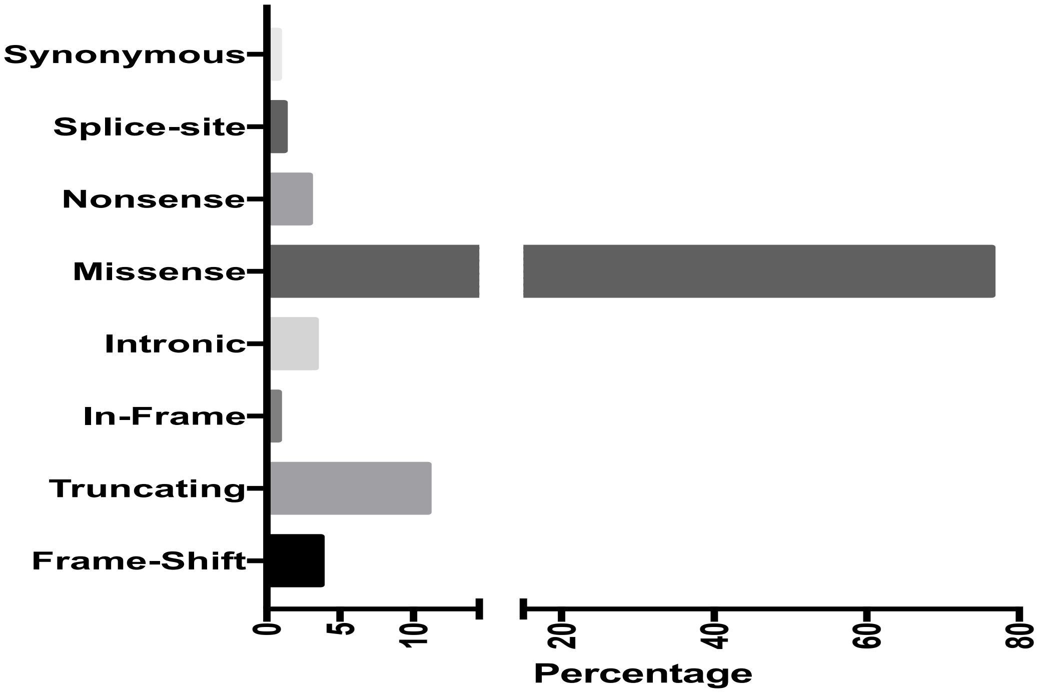

Frame-Shift	3.66
Truncating	10.97
Inframe	0.81
Intronic	3.25
Missense	76.42
Nonesense	2.85
Splice-site	1.22
Synonymous	0.81

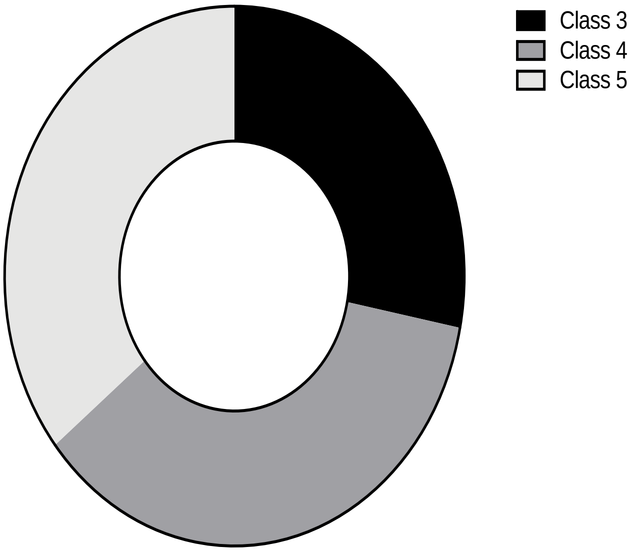

	%
Class 3	28.10
Class 4	36.20
Class 5	35.80

Figure S6 The percentage of mutations by class in the filtered sequencing output.

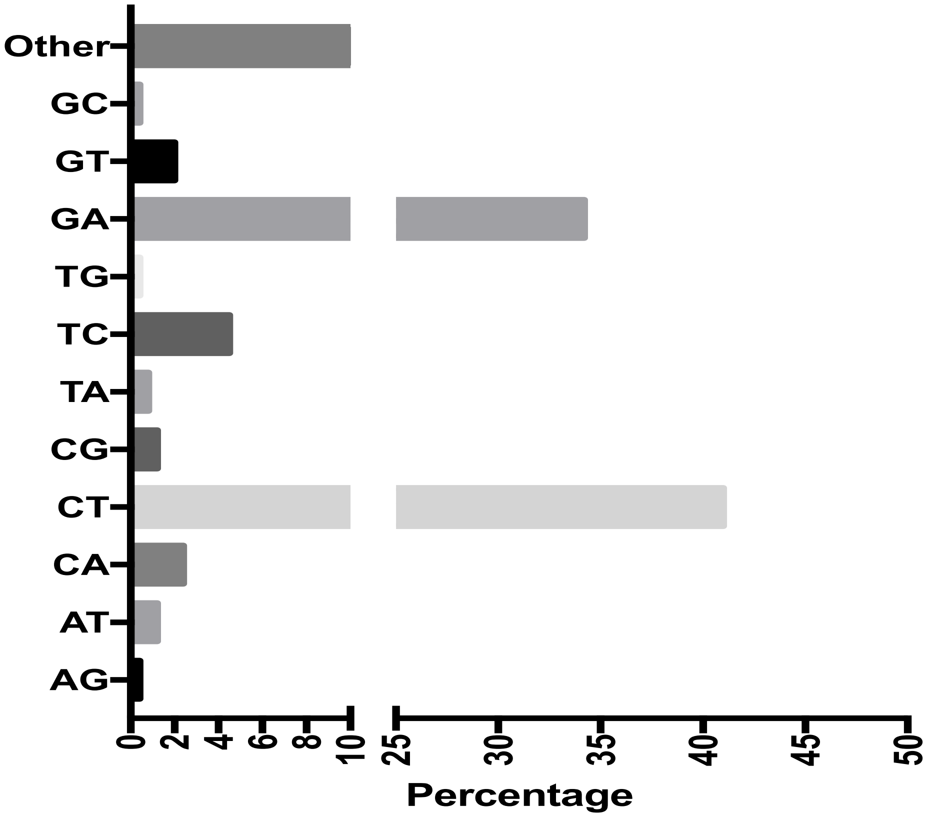

AG	0.4
AT	1.2
CA	2.4
CT	41
CG	1.2
TA	0.81
TC	4.5
TG	0.4
GA	34.2
GT	2
GC	0.4
Other	11.4

Figure S7 The percentage base changes in the filtered sequencing output.

4 Somatic Landscape by path_MMR gene

		path_MLH1 (%)	path_MSH2 (%)	path_MSH6 (%)	path_PMS2 (n)
MAPK Signalling	*KRAS*	21.43	24.14	12	1
	NRAS	0.00	3.45	6	0
	HRAS	0.00	3.45	0	0
	MAP2K1	7.14	3.45	0	0
	MAP2K2	7.14	3.45	0	0
	MAP2K4	0.00	3.45	0	0
	MAP3K1	7.14	3.45	12	0
	BRAF	14.29	3.45	0	0
	ARAF	21.43	3.45	41	0
	PTPN11	0.00	0.00	0	0
	GNAQ	7.14	3.45	0	0
PI(3)K Signalling	*PTEN*	57.14	68.97	59	1
	PIK3CA	28.57	31.03	41	2
	AKT1	7.14	0.00	12	0
	CARD11	0.00	0.00	0	0
	SRC	7.14	0.00	0	0
TGF-B Signalling	*SMAD4*	0.00	13.79	6	0
WNT/β-catenin	*APC*	0.00	20.69	0	0
	CDH1	0.00	3.45	0	0
	CTNNB1	7.14	10.34	12	1
Histone	*H3F3A*	0.00	0.00	6	0
	H3F3B	0.00	3.45	0	0
	EZH2	0.00	0.00	0	0
Proteolysis	*FBXW7*	0.00	17.24	12	0
Metabolism	*IDH1*	0.00	0.00	0	0
	IDH2	0.00	0.00	0	0
	GNAS	0.00	3.45	6	0
Genome integrity	*TP53*	14.29	24.14	12	1
	MDM2	0.00	3.45	0	0
	ATM	0.00	3.45	0	0
	MUTYH	0.00	3.45	6	1
	POLE	0.00	3.45	12	0
	MLH1	0.00	3.45	0	0
	KIT	7.14	6.90	0	0
	EGFR	0.00	0.00	12	0
Protien phosphatase	*NOTCH1*	14.29	3.45	0	0
	KDR	14.29	0.00	0	0
	PTK2	7.14	0.00	0	0
	CDK4	0.00	0.00	0	0

		path_MLH1 (%)	path_MSH2 (%)	path_MSH6 (%)	path_PMS2 (%)
RTK signalling	*FLT3*	0.00	0.00	6	0
	ERBB3	0.00	3.45	0	0
	ERBB2	14.29	3.45	12	0
	ERBB4	7.14	3.45	0	0
	FGFR3	0.00	3.45	6	0
	FGFR2	0.00	3.45	12	0
	PDGFRA	0.00	3.45	0	0
	PDGFRB	0.00	0.00	0	0
	ALK	0.00	0.00	0	0
	CDKN2A	0.00	0.00	6	0
Cell Cycle	*ABL1*	0.00	0.00	6	0
	CCND1	0.00	0.00	0	0
	CSF1R	0.00	0.00	0	0
	FGFR1	0.00	0.00	0	0
	JAK2	0.00	0.00	0	0
	JAK3	0.00	3.45	0	0
	NPM1	0.00	0.00	0	0
	GNA11	0.00	0.00	0	0
	MPL	0.00	0.00	0	0
	RB1	0.00	0.00	0	0
	PPP2R1A	0.00	3.45	0	0
	VHL	0.00	3.45	6	0
Transcription factor	*CIC*	0.00	0.00	12	0
	MED12	0.00	0.00	0	0
	MET	0.00	3.45	6	0
	RET	7.14	3.45	0	0
	SMARCB1	14.29	0.00	0	0
	SMO	14.29	3.45	0	0
	FOXL2	0.00	0.00	0	0
	MYC	0.00	0.00	0	0
	HNF1A	0.00	0.00	0	0
	EIF1AX	0.00	0.00	0	0
Tor Signalling	*STK11*	7.14	3.45	6	0
Immune processes	*MYD88*	7.14	0.00	0	0
	CD79A	7.14	0.00	0	0
	CD79B	7.14	0.00	0	0

Table S3 Somatic mutations in genes covered by The Leiden Endometrial onco-panel found in LS broken down by the germline pathogenic variation

5 A comparison of somatic landscape between path_*MLH1* vs somatic MSI-H *MLH1* promoter hyper-methylation

	Gene	Lynch MLH1 only (%)	*MLH1* Methylated (%)	P-value
MAPK Signalling	*KRAS*	21	39	0.2
	NRAS	0	7	0.3
	HRAS	0	2	0.6
	MAP2K1	7	2	0.3
	MAP2K2	7	0	0.05
	MAP2K4	0	5	0.4
	MAP3K1	7	8	0.9
	BRAF	14	0	0.005
	ARAF	21	5	0.05
	PTPN11	0	2	0.6
	GNAQ	7	2	0.3
PI(3)K Signalling	*PTEN*	57	88	0.007
	PIK3CA	29	51	0.2
	AKT1	7	5	0.77
	CARD11	0	8	0.3
	SRC	7	2	0.3
TGF-B Signalling	*SMAD4*	0	0	1
WNT/β-catenin	*APC*	0	14	0.2
	CDH1	0	5	0.4
	CTNNB1	7	20	0.25
Histone	*H3F3A*	0	2	0.6
	H3F3B	0	0	1
	EZH2	0	0	1
Proteolysis	*FBXW7*	0	8	0.3
Metabolism	*IDH1*	0	2	0.6
	IDH2	0	2	0.6
	GNAS	0	8	0.3
Genome integrity	*TP53*	14	8	0.5
	MDM2	0	2	0.6
	ATM	0	10	0.2
	MUTYH	0	3	0.5
	POLE	0	5	0.4
	MLH1	NA	3	NA
	KIT	7	3	0.5
	EGFR	0	2	0.6
RTK signalling	*FLT3*	0	3	0.5
	ERBB3	0	5	0.5
	ERBB2	14	7	0.4
	ERBB4	7	3	0.5
	FGFR3	0	0	1
	FGFR2	0	15	0.07
	PDGFRA	0	2	0.6
	PDGFRB	0	10	0.1
	ALK	0	8	0.3
	CDKN2A	0	0	1

	Gene	Lynch MLH1 only (%)	*MLH1* Methylated (%)	P-value
Cell Cycle	*ABL1*	0	7	0.3
	CCND1	0	14	0.1
	CSF1R	0	3	0.5
	FGFR1	0	2	0.6
	JAK2	0	5	0.4
	JAK3	0	2	0.6
	NPM1	0	0	1
	GNA11	0	2	0.6
	MPL	0	2	0.6
	RB1	0	8	0.3
	PPP2R1A	0	8	0.3
	VHL	0	0	1
Transcription factor	*CIC*	0	15	0.07
	MED12	0	8	0.3
	MET	0	5	0.4
	RET	7	5	0.8
	SMARCB1	14	3	0.1
	SMO	14	0	0.005
	FOXL2	0	0	1
	MYC	0	2	0.6
	HNF1A	0	5	0.4
	EIF1AX	0	5	0.4
Tor Signalling	*STK11*	7	2	0.3
Immune processes	*MYD88*	7	0	0.05
	CD79A	7	0	0.05
	CD79B	7	0	0.05
Protien phosphatase	*NOTCH1*	14	5	0.2
	KDR	14	2	0.05
	PTK2	7	2	0.3
	CDK4	0	2	0.6

Table S4 Somatic mutations in genes covered by The Leiden Endometrial onco-panel found in path_MLH1

6 Somatic landscape of Lynch cohort vs TCGA derived molecular cohorts

		LS Overall (%)	MLH1 Methylated (%)	POLE (%)	Copy number low (%)	Copy Number High (%)
MAPK Signalling	*KRAS*	20	39	50	15	8
	NRAS	3	7	21	1.1	1.7
	HRAS	2	2	0	1.1	5
	MAP2K1	3	2	7	0	1.7
	MAP2K2	3	0	7	0	5
	MAP2K4	2	5	14	0	1.7
	MAP3K1	6	8	71	2.3	0
	BRAF	5	0	29	0	5
	ARAF	17	5	36	0	8
	PTPN11	0	2	14	0	5
	GNAQ	3	2	7	0	0
PI(3)K Signalling	*PTEN*	61	88	93	76	17
	PIK3CA	34	51	71	54	60
	AKT1	5	5	7	1.1	7
	CARD11	0	8	50	1.1	5
	SRC	2	2	7	0	8
TGF-B Signalling	*SMAD4*	8	0	36	0	3
WNT/β-catenin	*APC*	9	14	86	2.3	1.7
	CDH1	2	5	36	2.3	0
	CTNNB1	11	20	36	54	3
Histone	*H3F3A*	2	2	14	3	8
	H3F3B	2	0	0	1.1	15
	EZH2	0	0	57	1.1	5
Proteolysis	*FBXW7*	11	8	79	6	22
Metabolism	*IDH1*	0	2	7	0	3
	IDH2	0	2	14	0	8
	GNAS	3	8	21	1.1	7
Genome integrity	*TP53*	20	8	36	1.1	92
	MDM2	2	2	7	0	0
	ATM	2	10	86	8	3
	MUTYH	5	3	7	0	1.7
	POLE	5	5	100	3	3
	MLH1	2	3	21	0	0
	KIT	5	3	71	0	3
	EGFR	3	2	29	1.1	1.7
RTK signalling	*FLT3*	2	3	43	0	0
	ERBB3	2	5	29	3	13
	ERBB2	8	7	14	1.1	25
	ERBB4	3	3	64	2.3	7
	FGFR3	3	0	14	0	10
	FGFR2	5	15	29	14	10
	PDGFRA	2	2	50	0	3
	PDGFRB	0	10	7	0	3
	ALK	0	8	43	1.1	13
	CDKN2A	2	0	0	1.1	3

		LS Overall (%)	MLH1 Methylated (%)	POLE (%)	Copy number low (%)	Copy Number High (%)
Cell Cycle	*ABL1*	2	7	7	1.1	0
	CCND1	0	14	21	7	7
	CSF1R	0	3	14	0	1.7
	FGFR1	0	2	29	1.1	15
	JAK2	0	5	57	0	5
	JAK3	2	2	36	0	23
	NPM1	0	0	14	0	0
	GNA11	0	2	7	1.1	3
	MPL	0	2	29	0	7
	RB1	0	8	64	1.1	7
	PPP2R1A	2	8	29	1.1	23
	VHL	3	0	7	1.1	3
Transcription factor	*CIC*	3	15	29	1.1	3
	MED12	0	8	7	7	1.7
	MET	3	5	29	0	3
	RET	3	5	64	1.1	0
	SMARCB1	3	3	57	1.1	3
	SMO	5	0	14	1.1	3
	FOXL2	0	0	0	0	8
	MYC	0	2	14	3	25
	HNF1A	0	5	7	1.1	5
	EIF1AX	0	5	7	0	1.7
Tor Signalling	*STK11*	5	2	7	0	3
Immune processes	*MYD88*	2	0	14	0	0
	CD79A	2	0	7	0	1.7
	CD79B	2	0	14	0	8
Protien phosphatase	*NOTCH1*	5	5	21	1.1	1.7
	KDR	3	2	71	0	1.7
	PTK2	2	2	36	1.1	17
	CDK4	0	2	7	0	0

Table S5 A comparison of somatic mutations in genes covered by The Leiden Endometrial onco-panel found in our LS cohort vs. the molecular cohorts as taken from the Cancer Genome Atlas

7 Effect of type of mutation: driver vs passenger

Genes of Interest

ABL1	*FGFR2*	*MLH1*
AKT1	*FGFR3*	*MPL*
ALK	*FLT3*	*MUTYH*
APC	*FOXL2*	*MYC*
ARAF	*GNA11*	*MYD88*
ATM	*GNAQ*	*NOTCH1*
BRAF	*GNAS*	*NPM1*
CARD11	*H3F3A*	*NRAS*
CCND1	*H3F3B*	*PDGFRA*
CD79A	*HNF1A*	*PDGFRB*
CD79B	*HRAS*	*PIK3CA*
CDH1	*IDH1*	*POLE*
CDK4	*IDH2*	*PPP2R1A*
CDKN2A	*JAK2*	*PTEN*
CIC	*JAK3*	*PTK2*
CSF1R	*KDR*	*PTPN11*
CTNNB1	*KIT*	*RB1*
EGFR	*KRAS*	*RET*
EIF1AX	*MAP2K1*	*SMAD4*
ERBB2	*MAP2K2*	*SMARCB1*
ERBB3	*MAP2K4*	*SMO*
ERBB4	*MAP3K1*	*SRC*
EZH2	*MDM2*	*STK11*
FBXW7	*MED12*	*TP53*
FGFR1	*MET*	*VHL*

Table S6 Genes included in the analysis

ALK	*GNA11*	*MYC*
CARD11	*H3F3A*	*NPM1*
CDK4	*HNF1A*	*PDGFRB*
CSF1R	*IDH1*	*PTPN11*
EZH2	*JAK2*	*RB1*
FGFR1	*MED12*	
FOXL2	*MPL*	

Table S7 Genes with no mutations in the Lynch cohort

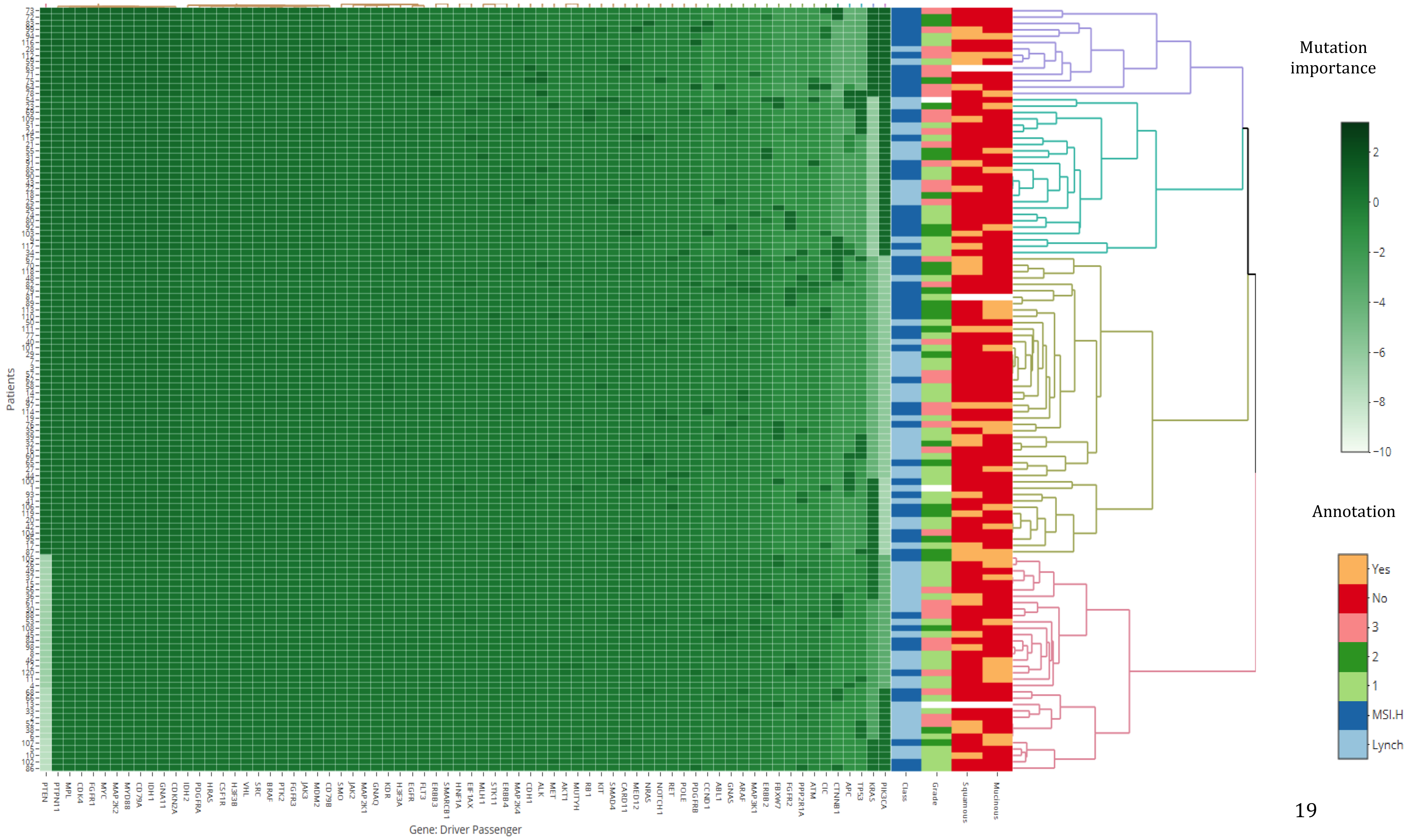

Patients
Gene: Driver Passenger
Class
Grade
Squamous
Mucinous
Mutation importance
2
0
-2
-4
-6
-8
-10
Annotation
Yes
No
3
2
1
MSI.H
Lynch

Figure S8 Mutation signatures within Lynch & MSI-H patients

A gene panel of 75 genes classified by “driver or passenger mutation” for 61 Lynch and 59 MSI-H patients. Clinical annotations for Class, Grade, Squamous, and Mucinous are provided as the rightmost columns. Intensities represent standardised and scaled signatures per patient observations (rows). Four principal dendrogrammatic clusters of genetic mutational signatures were observed indicative of genomic assault substructure within the pathologies. Akin to Figure 1 (scored “ever mutation”); gene clustering present mutations in *PTEN* (black triangle); *PIK3CA*, and *KRAS* (grey triangles) were the most important events in the two pathologies. Interestingly the peach dendrogram) cluster presents wild type *PTEN* associates predominantly with Lynch syndrome (light blue “Class” annotation). Concordant mutations in PTEN; PIK3CA, and KRAS predict predominantly an MSI-H phenotype (dark blue “Class” annotation; purple dendrogram). No associations were observed between the mutational signatures and disease grade, Squamous, or Mucinous status.

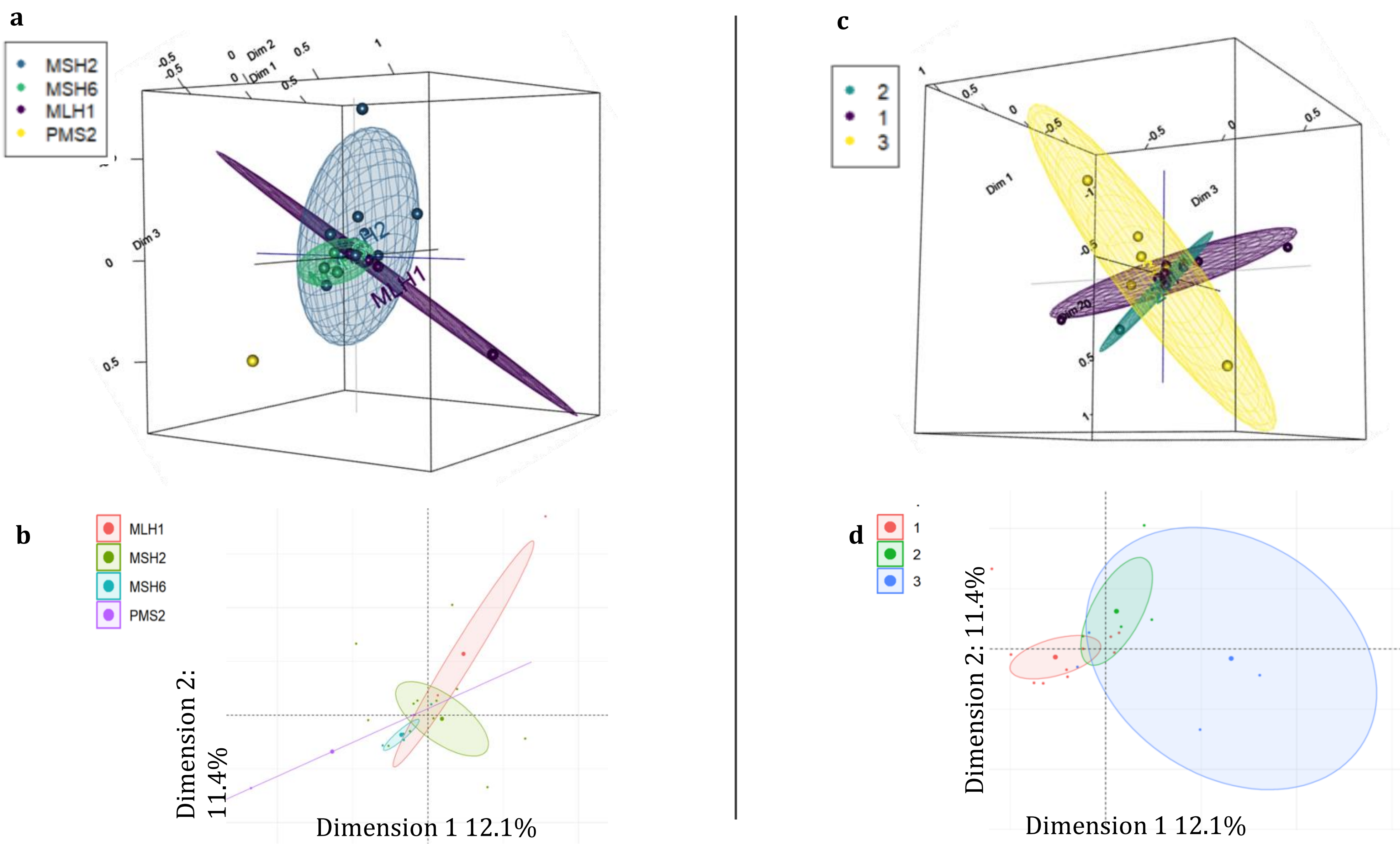
a
MSH2
MSH6
MLH1
PMS2
Dim 2
Dim 1
Dim 3
MLH1
b
MLH1
MSH2
MSH6
PMS2
Dimension 2: 11.4%
Dimension 1 12.1%
c
2
1
3
Dim 1
Dim 3
d
1
2
3
Dimension 2: 11.4%
Dimension 1 12.1%

Figure S9 Correspondence Analysis (MCA) of Lynch Syndrome patients: Genomic Profile & Grade

The 56 gene panel was subject to a focused analysis of 27 Lynch patients for which all clinical annotations were available. 3D MCA and associated 2D Biplots of Lynch patients are plotted by the high-fidelity ordinal "Ranked Severity" scoring matrix. Patients with similar profiles occupy close positions in 3D space (or 2D maps) whilst conversely negatively correlated gene mutation variables position patients at opposite sides of the plot origin. Mapped ellipsoids provide clinical annotations as 3D coverage over patient data points, wherein greater ellipsoidal definition represents tighter clustering of similar patients. Clinical annotations intersect one another and thus no distinct clinical measurements can be fully resolved based exclusively upon the mutational signatures within Lynch Syndrome. Of great interest each clinically annotated ellipsoid occupies a discrete orientation and spread within Euclidian space that strongly indicates the mutational signatures from Ranked Severity ordinal scoring can be related to distinct clinical Lynch Syndrome profiles.

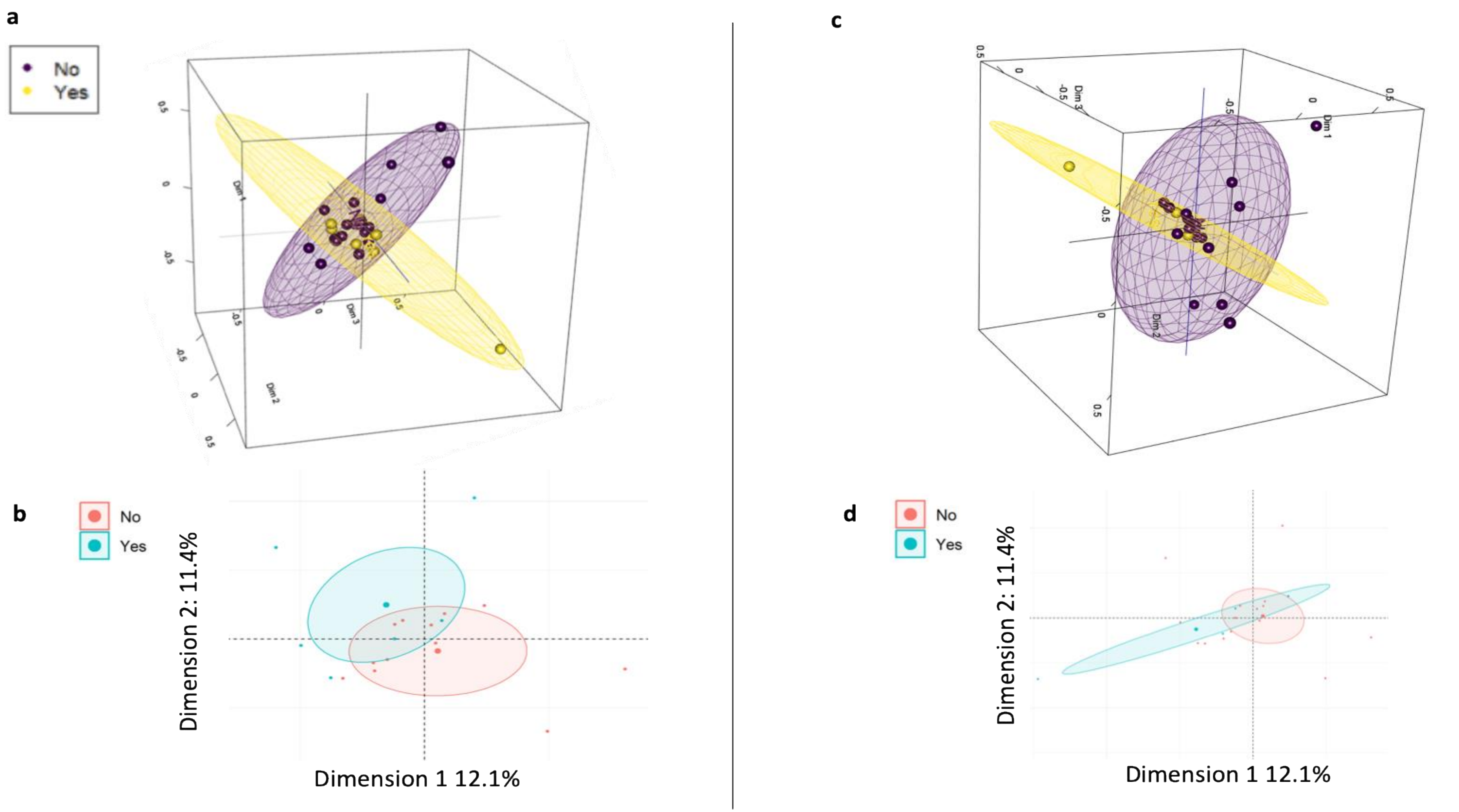
a
No
Yes
Dim 1
Dim 2
Dim 3
b
No
Yes
Dimension 2: 11.4%
Dimension 1 12.1%
c
Dim 1
Dim 2
Dim 3
d
No
Yes
Dimension 2: 11.4%
Dimension 1 12.1%

Figure S10 MCA of Lynch syndrome patients: Mucinous and Squamous status

The 56 gene panel was subject to a focused analysis of 27 Lynch patients for which all clinical annotations were available. 3D MCA and associated 2D Biplots of Lynch patients are plotted by the high fidelity ordinal "Ranked Severity" scoring matrix. Patients with similar profiles occupy close positions in 3D space (or 2D maps) whilst conversely negatively correlated gene mutation variables position patients at opposite sides of the plot origin. Mapped ellipsoids provide clinical annotations as 3D coverage over patient data points, wherein greater ellipsoidal definition represents tighter clustering of similar patients. Clinical annotations intersect one another and thus no distinct clinical measurements can be fully resolved based exclusively upon the mutational signatures within Lynch Syndrome. Of great interest each clinically annotated ellipsoid occupies a discrete orientation and spread within Euclidian space that strongly indicates the mutational signatures from Ranked Severity ordinal scoring can be related to distinct clinical Lynch Syndrome profiles.

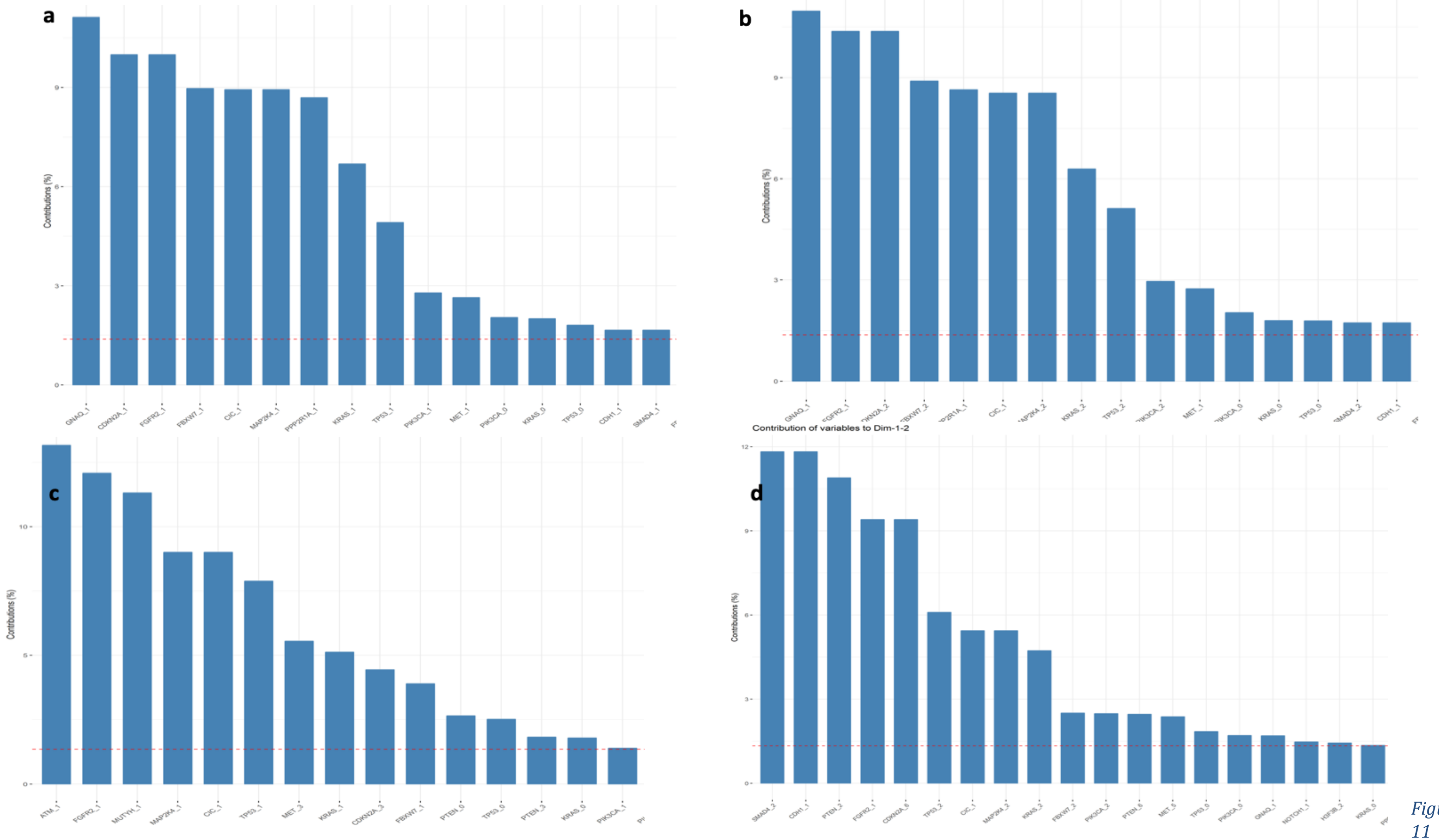

Figure 11

Figure S11 Contribution of gene mutations to MCA dimensions 1&2 for Lynch syndrome

A gene panel of 56 genes for 27 Lynch patients following removal of missing not at random clinical data. A). Binary "Ever mutation". B). Ordinal Driver| Passenger. C). Mutation Type. D). Ranked Severity. Gene mutations with the largest values contribute the most to the definition of the dimensions and Dimension 1 and 2 are the most important in explaining the variability in the data. The red dashed line represents the expected average value under the null hypothesis; thus gene mutations above this line contribute more than would be expected under the null hypothesis.

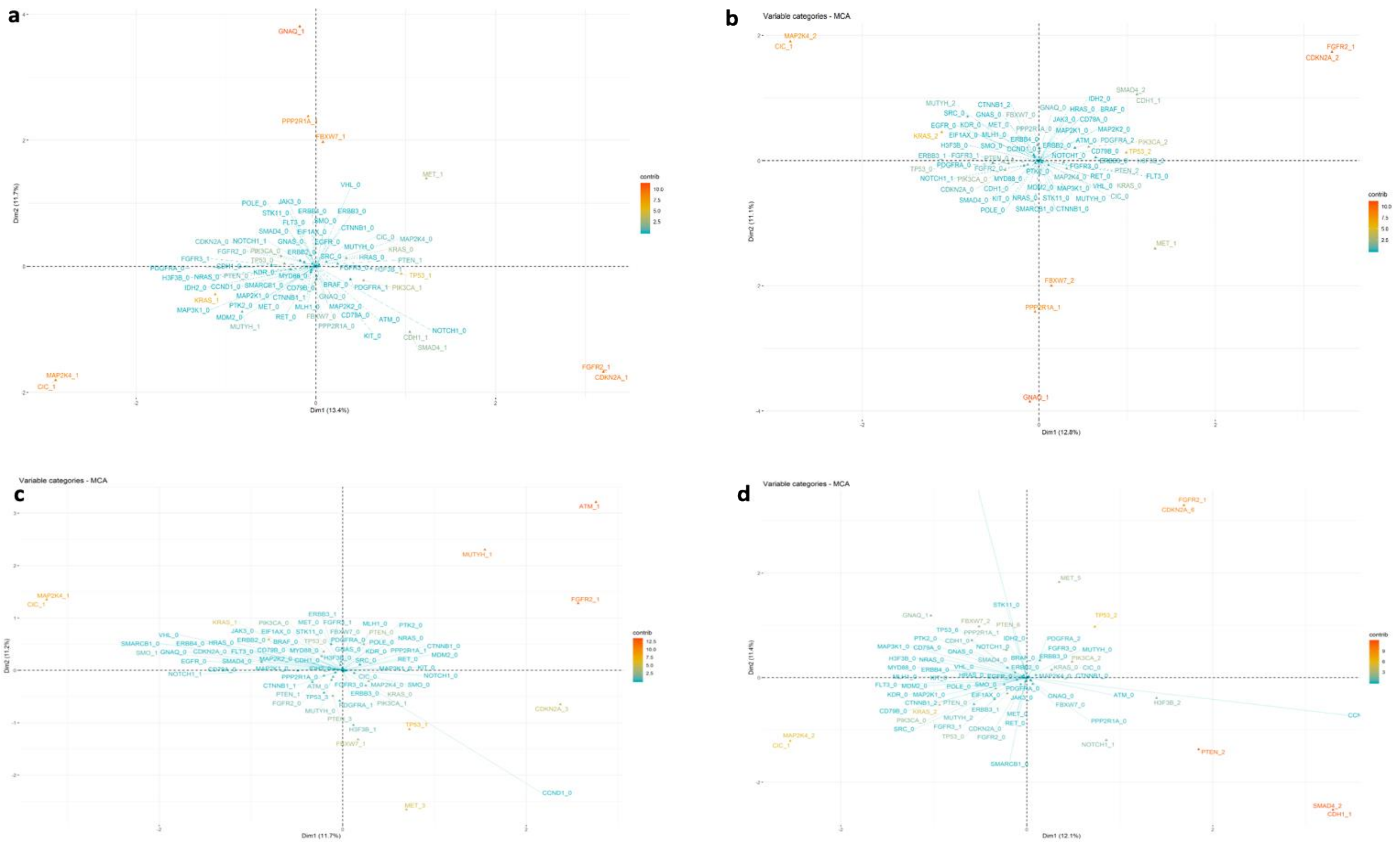

Figure S12 Biplot variable contribution of gene mutations to MCA dimensions 1 & 2 for Lynch syndrome

A gene panel of 56 genes for 27 Lynch patients following removal of missing not at random clinical data. A). Binary "Ever mutation". B). Ordinal Driver| Passenger. C). Mutation Type. D). Ranked Severity. Dimension 1 (x axis) is plotted against dimension 2 (y axis) with xy position representing the dimension(s) the gene contributes the most to.

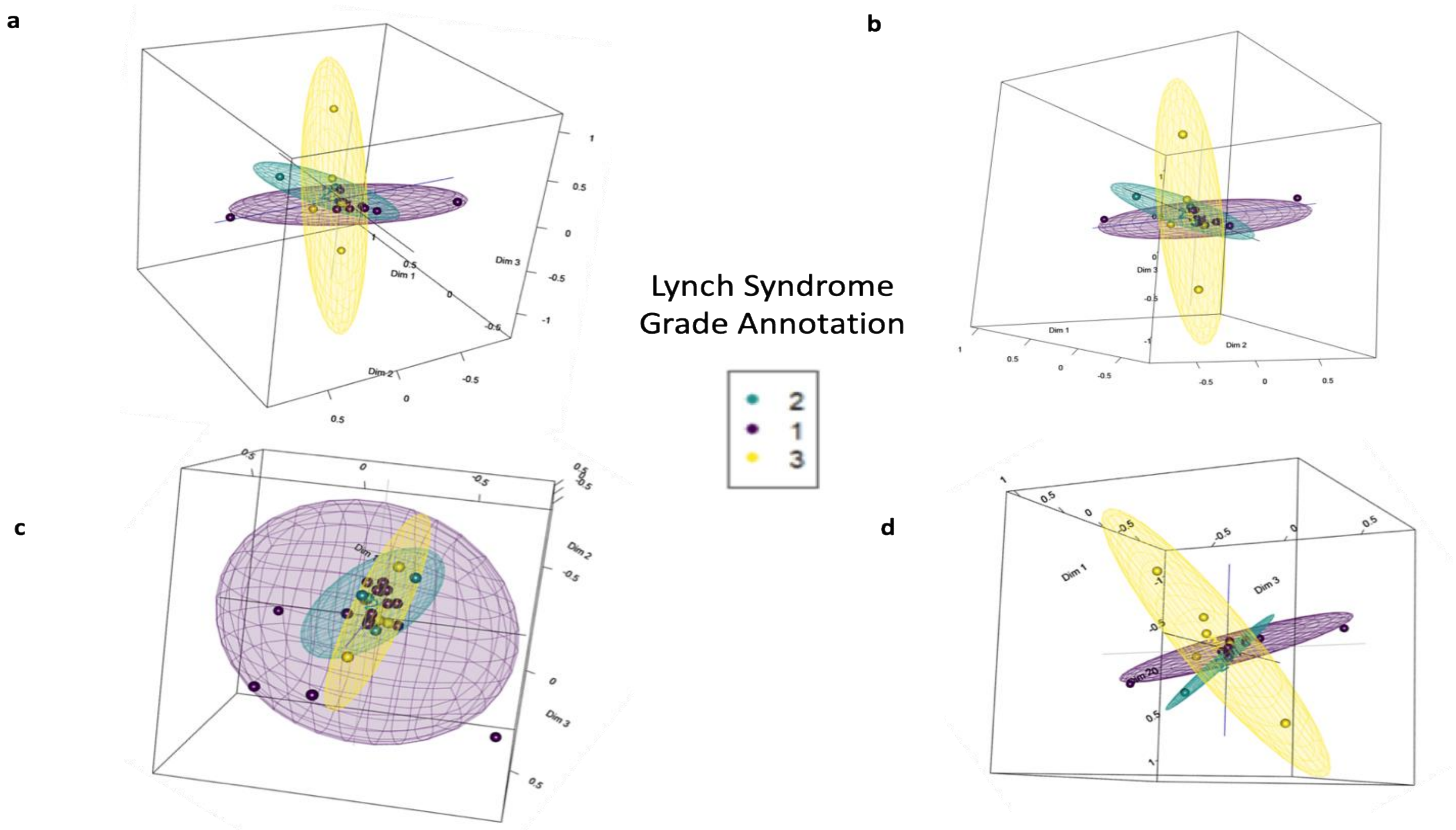

Figure S13 Representative comparison for four MCA 3D plots for Lynch syndrome "Grade" annotation for each of the four scoring matrices

A gene panel of 56 genes for 27 Lynch patients following removal of missing not at random clinical data. A). Binary “Ever mutation”. B). Ordinal Driver| Passenger. C). Mutation Type. D). Ranked Severity. Distances between points provide a measure of similarity / dissimilarity wherein points with similar profiles occupy close positions in 3D space (or 2D maps) whilst conversely negatively correlated gene mutation variables position at opposite sides of the plot origin. Ranked Severity (d) ordinal scoring provides the highest fidelity matrix, which is reflected in the definition of the ellipsoids for each of the three Grade classes, in contrast to **a**,**b**, or **c**
